# BRD4/MAP2K7/PGF Signaling Axis Promotes Senescence and Extracellular Matrix Metabolism of Nucleus Pulposus Cells in Intervertebral Disk Degeneration

**DOI:** 10.1111/acel.70034

**Published:** 2025-03-25

**Authors:** Guangzhi Zhang, Lei Li, Zhili Yang, Zhenyu Cao, Xuchang Hu, Yonggang Wang, Xuewen Kang

**Affiliations:** ^1^ Department of Orthopedics Lanzhou University Second Hospital Lanzhou Gansu People's Republic of China; ^2^ The Second Clinical Medical College Lanzhou University Lanzhou Gansu People's Republic of China; ^3^ Key Laboratory of Orthopedics Disease of Gansu Province Lanzhou University Second Hospital Lanzhou Gansu Province People's Republic of China; ^4^ The International Cooperation Base of Gansu Province for the Pain Research in Spinal Disorders Lanzhou Gansu Province People's Republic of China

**Keywords:** BRD4, extracellular matrix, intervertebral disk degeneration, nucleus pulposus, senescence

## Abstract

Intervertebral disk degeneration (IDD) is a common age‐related degenerative disease of the spine that imposes a substantial economic burden on both families and society. Despite substantial advances in understanding the mechanisms underlying IDD, effective therapeutic interventions for its treatment and prevention remain elusive. Our previous study identified a positive correlation between IDD severity and bromodomain‐containing protein 4 (BRD4) expression. However, the multifaceted role of BRD4 in IDD is still not fully understood. This study explored the abnormal elevation of BRD4 expression in nucleus pulposus (NP) tissues from patients with IDD and in an age‐related rat model of IDD. We found that BRD4 levels were positively correlated with NP senescence and extracellular matrix (ECM) degradation and inversely correlated with ECM anabolism. These relationships were further confirmed through assays measuring senescence‐associated β‐galactosidase activity, the expression of senescence markers P21 and P16, senescence‐associated secretory phenotype indicators (IL‐6, IL‐8, MMP3, and MMP13), as well as ECM metabolism markers such as collagen II and aggrecan. Mechanistically, aberrant BRD4 expression was found to upregulate MAP2K7, which in turn enhances PGF expression, promoting NP cell senescence and ECM metabolism. These findings highlight the crucial role of the BRD4/MAP2K7/PGF signaling axis in cellular senescence and ECM regulation, suggesting that BRD4 represents a promising therapeutic target for IDD.

## Graphical abstract

This study identifies BRD4 as a key regulator in intervertebral disk degeneration (IDD), linking its upregulation to NP cell senescence and disrupted ECM metabolism through the BRD4/MAP2K7/PGF signaling cascade, offering a potential therapeutic target for IDD treatment.

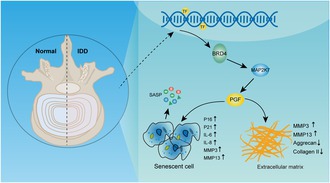

## 1 Introduction

Intervertebral disk degeneration (IDD) is a common age‐related degenerative spinal disorder that significantly impairs patients' occupational function and quality of life, while imposing substantial economic burdens on both families and society (Ran et al. 2024; Farrag et al. 2024). Research suggests that approximately 40% of individuals under the age of 30 years are affected by IDD, whereas nearly 90% of those aged 55 years and older experience moderate‐to‐severe manifestations (Ohnishi et al. 2018). As IDD progresses chronically, it may lead to various spine‐related pathologies, including disk herniation, spondylolisthesis, spinal stenosis, and degenerative scoliosis (Azril et al. 2023; Martin et al. 2019). These conditions often manifest as acute or chronic lower back pain (LBP), a widespread public health concern worldwide, with around 40% of LBP cases attributed to IDD (Martin et al. 2019). Despite its high prevalence, the precise mechanisms driving IDD remain incompletely understood. A more comprehensive elucidation of the molecular pathways involved in IDD progression could facilitate the identification of biomarker‐driven therapeutic targets, enabling the development of personalized treatment strategies.

Cellular senescence is characterized by the irreversible cessation of cell proliferation, with the cell cycle permanently arrested in the G0/G1 phase (Ogrodnik et al. 2024; Cho and Kim 2024). Senescent cells exhibit distinctive morphological features, including increased size, cytoplasmic accumulation of pigments and vacuoles, enlarged nuclei, nuclear membrane invagination, and chromatin condensation, ultimately leading to cellular demise (Chen et al. 2022; Yan et al. 2019). During senescence, the expression of cell cycle inhibitory proteins such as P53, P21, and P16 is upregulated, along with an increase in senescence‐associated β‐galactosidase (SA‐β‐gal) activity. These molecular changes serve as primary markers for identifying cellular senescence (Tian et al. 2024). Another hallmark of senescent cells is their secretion of pro‐inflammatory factors (e.g., IL‐1α, IL‐1β, IL‐6, and IL‐8), growth factors (e.g., HGF, TGF‐β, and GM‐CSF), chemokines (e.g., CXCL‐1/3 and CXCL‐10), and matrix remodeling enzymes (e.g., matrix metalloproteinases [MMPs]), collectively known as the senescence‐associated secretory phenotype (SASP) (Tian et al. 2024; Noh et al. 2024). The roles and mechanisms of SASP have recently garnered attention, particularly in age‐related diseases such as IDD, osteoarthritis, and cancer, where they significantly influence disease onset and progression (Fujita et al. 2024). Increasing evidence links IDD to the senescence of nucleus pulposus (NP) cells, resulting in a decline in functional cells and the heightened release of inflammatory and chemotactic factors (Song, Liang, Li, et al. 2024; Ottone et al. 2022; Liu, Sun, Wang, et al. 2024). This process accelerates the senescence of nearby cells, contributing to dysfunction and structural damage within the intervertebral disk (Zhang et al. 2021). The physiological function of intervertebral disks is intrinsically associated with the molecular composition of the extracellular matrix (ECM) (Ohnishi et al. 2023). In healthy disks, a homeostatic balance between ECM synthesis and degradation is maintained. However, senescent NP cells disrupt this balance, decreasing ECM production and increasing its catabolism, leading to pathological changes in the ECM. As NP cell senescence progresses, there is an upregulation of inflammatory factors, chemokines, and matrix‐degrading enzymes such as MMP3, MMP9, and MMP13, which constitute the SASP (Wang et al. 2024; Gao et al. 2024). This upregulation reduces the synthesis of proteoglycans and type II collagen while gradually increasing the production of type I collagen (Zhang, Li, et al. 2023). However, the precise mechanisms governing NP cell senescence in the pathogenesis and progression of IDD remain poorly understood.

Bromodomain‐containing protein 4 (BRD4) is a member of the bromodomain and extra‐terminal (BET) family, functioning as a critical transcriptional and epigenetic regulator. BRD4 enhances RNA polymerase II activity by facilitating the assembly of protein complexes, thus stimulating both transcription initiation and elongation (Wei et al. 2024; Zhang and Roeder 2025). This process plays a crucial role in regulating various biological processes, including gene transcription, cell cycle progression, and inflammatory responses (Zhang et al. 2024; Song, Li, et al. 2024). Studies have linked BRD4 to a range of age‐related diseases, including IDD, osteoarthritis, cardiovascular diseases, and cancer (Xu et al. 2024; Lin and Du 2020). Recent research has increasingly focused on elucidating the role of BRD4 in the skeletal system, with particular attention to its involvement in IDD. Wang et al. (Wang et al. 2019) demonstrated that alterations in BRD4 expression influence ECM metabolic processes in NP cells, contributing to IDD progression, particularly in the context of diabetes. Our previous study revealed significant upregulation of BRD4 expression in severely degenerated NP tissues, suggesting a crucial role for BRD4 in the pathogenesis and progression of IDD (Zhang, Chen, et al. 2022). However, the precise functional implications of BRD4 in IDD have yet to be fully elucidated.

In this study, we observed elevated BRD4 expression in NP tissues from patients with IDD and in an age‐related rat model of IDD. This upregulation of BRD4 appears to be a key factor contributing to NP cell senescence and disrupted ECM metabolism, playing a crucial role in the pathogenesis of IDD. Mechanistically, BRD4 upregulates MAP2K7 expression, which subsequently activates the downstream expression of PGF. This BRD4/MAP2K7/PGF signaling cascade drives NP cell senescence and abnormal ECM metabolism, thus accelerating IDD progression. Collectively, our findings highlight the importance of BRD4 in the chronic progression of IDD and reveal a novel mechanistic paradigm in which the BRD4/MAP2K7/PGF signaling pathway mediates IDD progression. These insights provide a potential therapeutic target for the treatment of IDD.

## 2 Results

### 2.1 BRD4 Regulates Cellular Senescence and ECM Metabolism in NP Tissues of Patients with IDD

To elucidate the major biological functions mediated by BRD4, we constructed BRD4 co‐expressed gene networks using the STRING database and performed Gene Ontology (GO) and Kyoto Encyclopedia of Genes and Genomes (KEGG) enrichment analyses. In total, 45 genes were identified through co‐expression analyses (Figure S1a). GO enrichment analysis revealed significant enrichment in biological processes such as response to signal transduction by the p53 class mediator, positive regulation of the cell cycle, and regulation of fat cell proliferation. Significantly enriched cellular components included nuclear chromatin, ATPase complex, and the mitotic spindle midzone, whereas enriched molecular functions included lysine‐acetylated histone binding, ATP‐dependent DNA helicase activity, beta‐catenin binding, p53 binding, and NF‐kappaB binding. KEGG enrichment analysis identified enrichment in pathways related to cellular senescence, nucleotide excision repair, DNA replication, mismatch repair, and the cell cycle (Figure S1b,c; Table S1). Based on these findings, we propose that BRD4 plays a crucial role in the initiation and progression of NP cell senescence. As a result, our research has focused on uncovering the underlying mechanisms by which BRD4 regulates NP cell senescence in the context of IDD.

**FIGURE 1:**
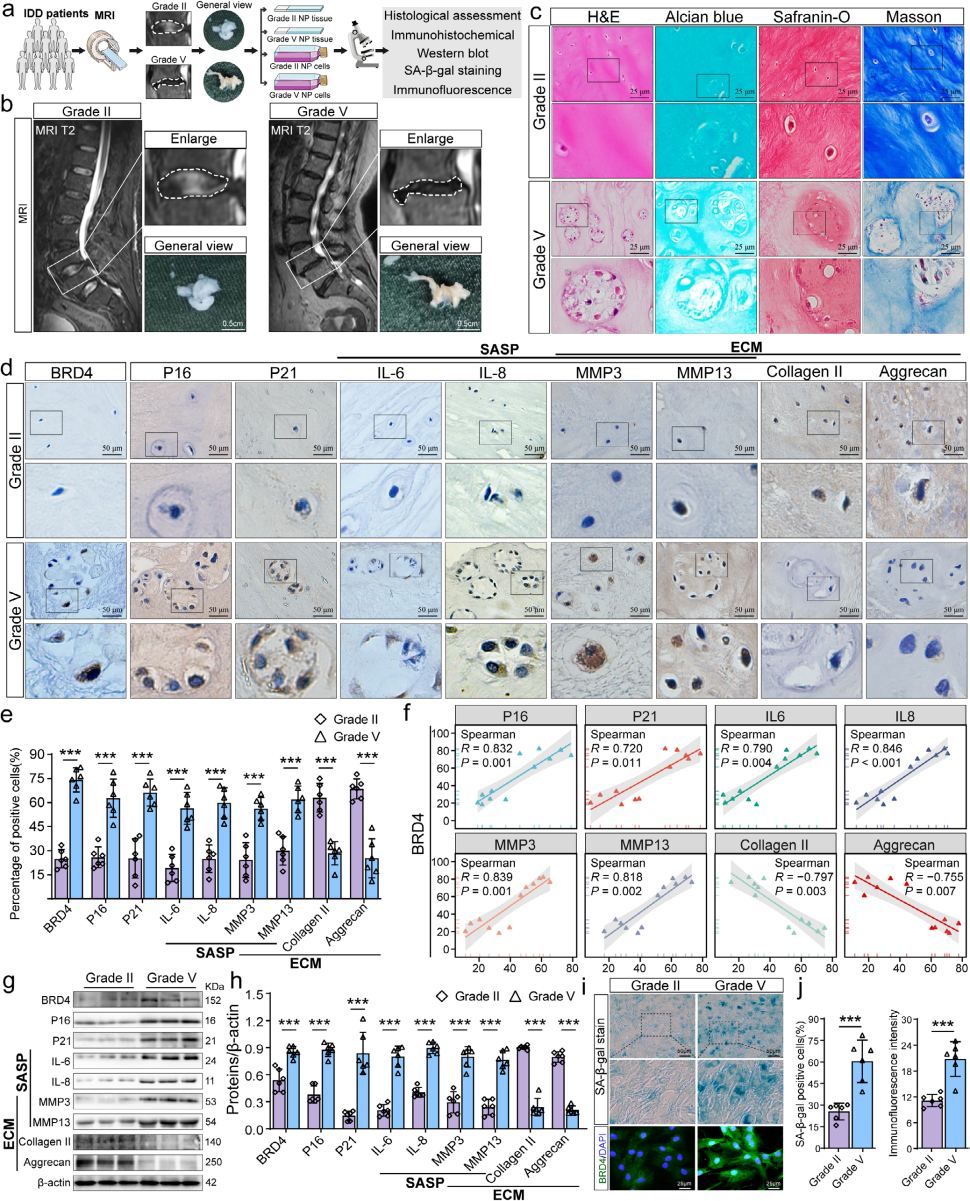
BRD4 regulates cellular senescence and ECM metabolism in NP tissues of patients with IDD. (a) Schematic representation of histological analysis combined with T2‐weighted MRI of the spine in patients with IDD. NP tissues with mild degeneration (Grade II) and severe degeneration (Grade V) according to the Pfirrmann grading system were collected. (b) Representative sagittal T2‐weighted MRI and gross morphological changes of NPs in patients with IDD from Grade II and V groups based on the Pfirrmann grading system. (c) Representative images stained with H&E, Safranin‐O, Alcian Blue, and Masson's trichrome stain of NP tissues from Grade II and V groups. (d, e) Representative immunohistochemical images and quantitative analysis of BRD4, senescence markers (P16 and P21), SASP markers (including IL‐6, IL‐8, MMP3, and MMP13), and ECM metabolism‐related markers (including MMP3, MMP13, collagen II, and aggrecan) in NP tissues of patients with IDD. (f) Correlation analysis of BRD4 expression changes with P16, P21, SASP markers, collagen II, and aggrecan based on immunohistochemical results. (g, h) Western blot and quantitative analysis of BRD4, P16, P21, IL‐6, IL‐8, MMP3, MMP13, collagen II, and aggrecan in two groups. (g, h) Representative images and quantitative analysis of SA‐β‐gal staining in NP cells cultured in vitro from Grade II and Grade V patients with IDD. (i, j) Representative images and quantitative analysis of BRD4 protein expression changes in NP cells from Grade II and V groups. Data are presented as mean ± SD. *p < 0.05, **p < 0.01, ***p < 0.001.

Using the Pfirrmann grading system for Magnetic Resonance Imaging (MRI) in IDD, we selected mildly degenerated NP tissues (Grade II) and severely degenerated NP tissues (Grade V) for histological analysis (Figure 1a). The Grade II group exhibited high water content, evidenced by a high signal on T2‐weighted MRI, and macroscopic examination revealed a white, gelatinous texture. Conversely, the Grade V group showed a reduced T2‐weighted MRI signal, indicating severe degeneration, with macroscopic examination revealing a hardened texture and yellow color (Figure 1b). Hematoxylin & eosin (H&E) staining showed a more organized arrangement of collagen fibers and deeper ECM staining in the Grade II group, with the presence of small vacuolated cells. In the Grade V group, there was a marked absence and disorganization of collagen fibers, lighter ECM staining, and numerous large vacuolated cells (Figure 1c). Additional staining techniques, including Safranin O, Alcian blue, and Masson's trichrome, indicated a significant decrease in proteoglycan and collagen fiber content in the Grade V group (Figure 1c).

Previous studies have demonstrated a strong correlation between IDD and NP cell senescence (Silwal et al. 2023; Kritschil et al. 2024; Ambrosio et al. 2024). Cellular senescence not only results in increased expression of senescence markers P16 and P21 but also leads to an elevated expression of SASP molecules, which further accelerate peripheral cellular senescence through autocrine and paracrine effects. These changes profoundly affect ECM metabolism, with decreased anabolic activity and increased catabolic activity (Zhang, Li, et al. 2023). To explore further the relationship between BRD4 expression, NP tissue senescence, and ECM metabolism, we performed immunohistochemistry. The results revealed a significant increase in BRD4 expression in the Grade V group. Additionally, there was a marked elevation in the expression of senescence markers P16 and P21, as well as SASP markers, including IL‐6, IL‐8, MMP3, and MMP13. Conversely, the expression of ECM markers such as collagen II and aggrecan decreased, whereas MMP3 and MMP13 expression increased (Figure 1d,e). Correlation analysis showed that BRD4 expression was positively correlated with P16, P21, IL‐6, IL‐8, MMP3, and MMP13, and negatively correlated with collagen II and aggrecan (Figure 1f). Western blot and immunohistochemistry results were consistent (Figure 1g,h). Furthermore, we conducted cell cultures on NP tissue samples collected from patients from Grade II and V groups. SA‐β‐gal staining revealed a substantial increase in NP cell senescence in the Grade V group compared with the Grade II group. Immunofluorescence analysis further demonstrated a significant increase in BRD4 expression in the Grade V group (Figure 1i,j).

### 2.2 BRD4 Regulates Cellular Senescence and ECM Metabolism in NP Tissues of Age‐Related IDD Models in SD Rats

**FIGURE 2:**
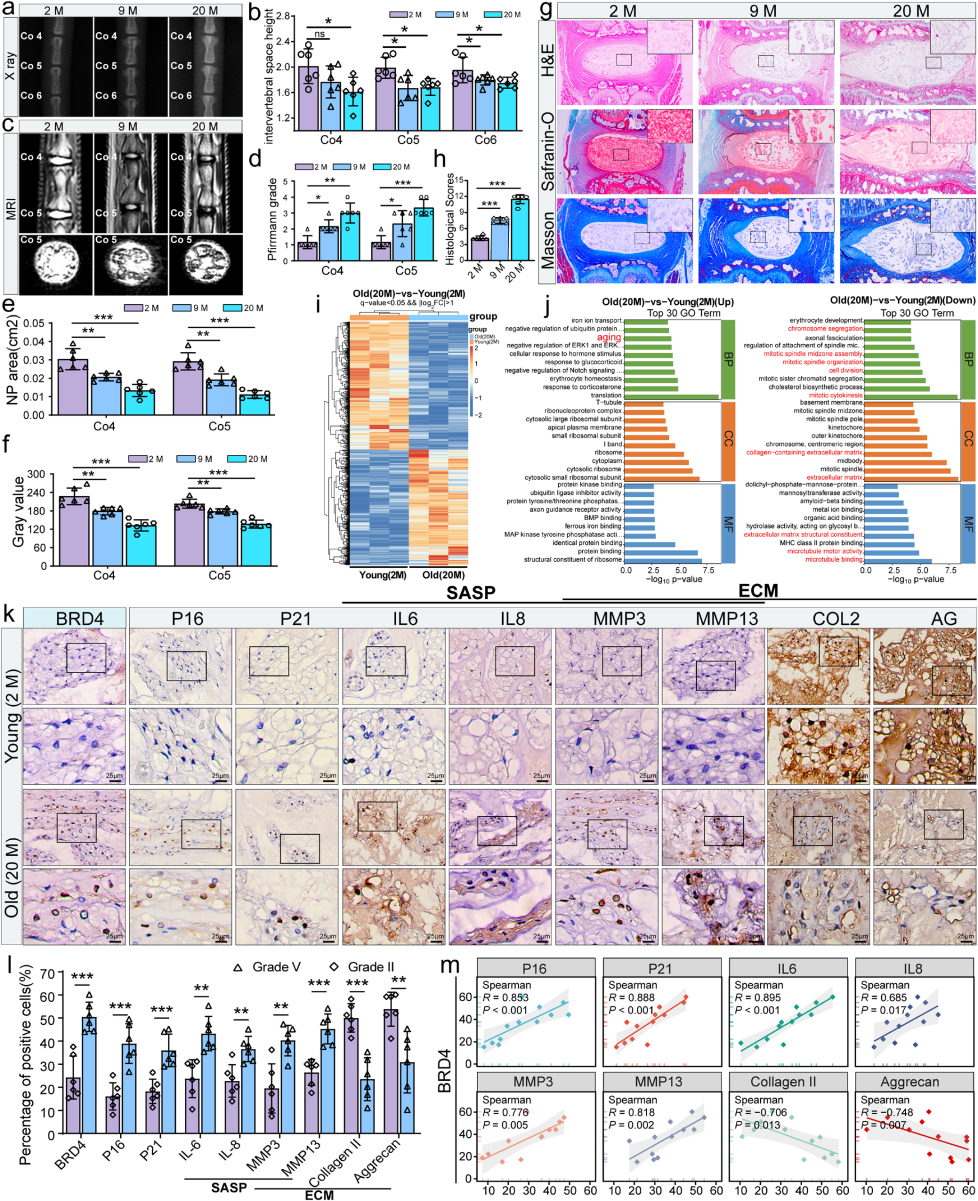
BRD4 regulates cellular senescence and ECM metabolism in NP tissues of age‐related IDD models in SD rats. (a, b) Representative X‐ray images and quantitative analysis of intervertebral disk height in the coccygeal vertebrae of rats at different ages (2 months [2 M], 9 months [9 M], and 20 months [20 M]). (c) Representative T2‐weighted MRI of the coccygeal vertebrae from rats in the various age groups. (d–f) Quantitative analysis of Pfirrmann grading, NP area, and gray value in T2‐weighted MRI across the different groups. (g) Representative histological images of coccygeal vertebrae from the different groups, stained with H&E, Safranin O‐Fast Green, and Masson's trichrome. (h) Quantitative analysis of histological scores based on H&E staining. (i) Heatmap depicting DEGs from transcriptomic sequencing of NP tissues between 2‐month‐old and 20‐month‐old rats. (j) GO enrichment analysis of the top 30 upregulated and downregulated genes. (k, l) Representative images and quantitative analysis of immunohistochemical staining, showing changes in the expression levels of BRD4, P16, P21, IL‐6, IL‐8, MMP3, MMP13, collagen II, and aggrecan in rat NP tissues. (m) Correlation analysis of BRD4 expression with P16, P21, IL‐6, IL‐8, MMP3, MMP13, collagen II, and aggrecan, based on the immunohistochemical statistical data. Results are presented as mean ± SD. *p < 0.05, **p < 0.01, ***p < 0.001, ns, not statistically significant.

Aging is a major risk factor for the chronic progression of IDD, with NP tissue degeneration increasing progressively with age in patients with IDD (Silwal et al. 2023; Liu, Sun, Zhang, et al. 2024). In this study, we established an age‐related rat model of IDD to investigate further changes in BRD4 expression and its relationship with cellular senescence and ECM metabolism. Sprague–Dawley (SD) rats aged 2 months (2 M), 9 months (9 M), and 20 months (20 M) were selected to assess the degree of IDD through imaging and histological evaluations. X‐rays revealed that the height of the caudal vertebral intervertebral space decreased in the 9 M and 20 M rats compared with the 2 M group, with a progressive reduction in height correlating with age (Figure 2a,b). MRI analysis confirmed this correlation, as the Pfirrmann grading of the coccygeal vertebrae under T2‐weighted imaging showed a gradual increase with age. Additionally, there was a progressive reduction in both the area of NP tissue and the grayscale value (Figure 2c–f). Furthermore, H&E staining showed age‐related degenerative changes, characterized by a loss of distinction between the NP and the annulus fibrosus, reduced ECM production, and NP cell accumulation. These changes were most prominent in the 20 M rats (Figure 2g). Furthermore, there was a significant increase in the histological scores of the intervertebral disks, which directly correlated with advancing age (Figure 2h). Saffron O, Alcian blue, and Masson staining further indicated a marked decrease in collagen and proteoglycan content in the NP tissue with increasing age of rats (Figure 2). In summary, our study confirmed the development of age‐related IDD in rats through imaging and histological analyses. Moreover, we successfully established an age‐associated IDD model, with rats aged 20 months demonstrating the most pronounced degenerative changes. Subsequently, we obtained NP tissues from rats aged 2 M and 20 M for transcriptomic sequencing. This analysis revealed significant gene expression alterations between the two groups. A heatmap illustrated distinct gene expression profiles (Figure 2i). GO analysis indicated that these differentially expressed genes were predominantly involved in NP cell senescence and ECM processes, strongly supporting the link between aging and IDD at the genetic level (Figure 2j). To elucidate further the relationship between BRD4 expression and NP tissue senescence and ECM metabolism, we conducted immunohistochemical analysis. The results showed a significant increase in BRD4 expression in the NP tissues of 20 M rats. Simultaneously, there was a marked elevation in the expression of senescence markers P16 and P21, as well as SASP indicators. Conversely, the expression of ECM anabolic markers, such as collagen II and aggrecan, decreased, whereas the expression of catabolic markers MMP3 and MMP13 increased (Figure 2k,l). BRD4 expression was positively correlated with P16, P21, IL‐6, IL‐8, MMP3, and MMP13, while it exhibited an inverse correlation with collagen II and aggrecan (Figure 2m). Western blot analysis confirmed these findings, showing results consistent with the immunohistochemical analysis (Figure S2a,b).

### 2.3 BRD4 Is Upregulated in Senescent Cell Models and Mediates NP Cell Senescence and ECM Metabolism

**FIGURE 3:**
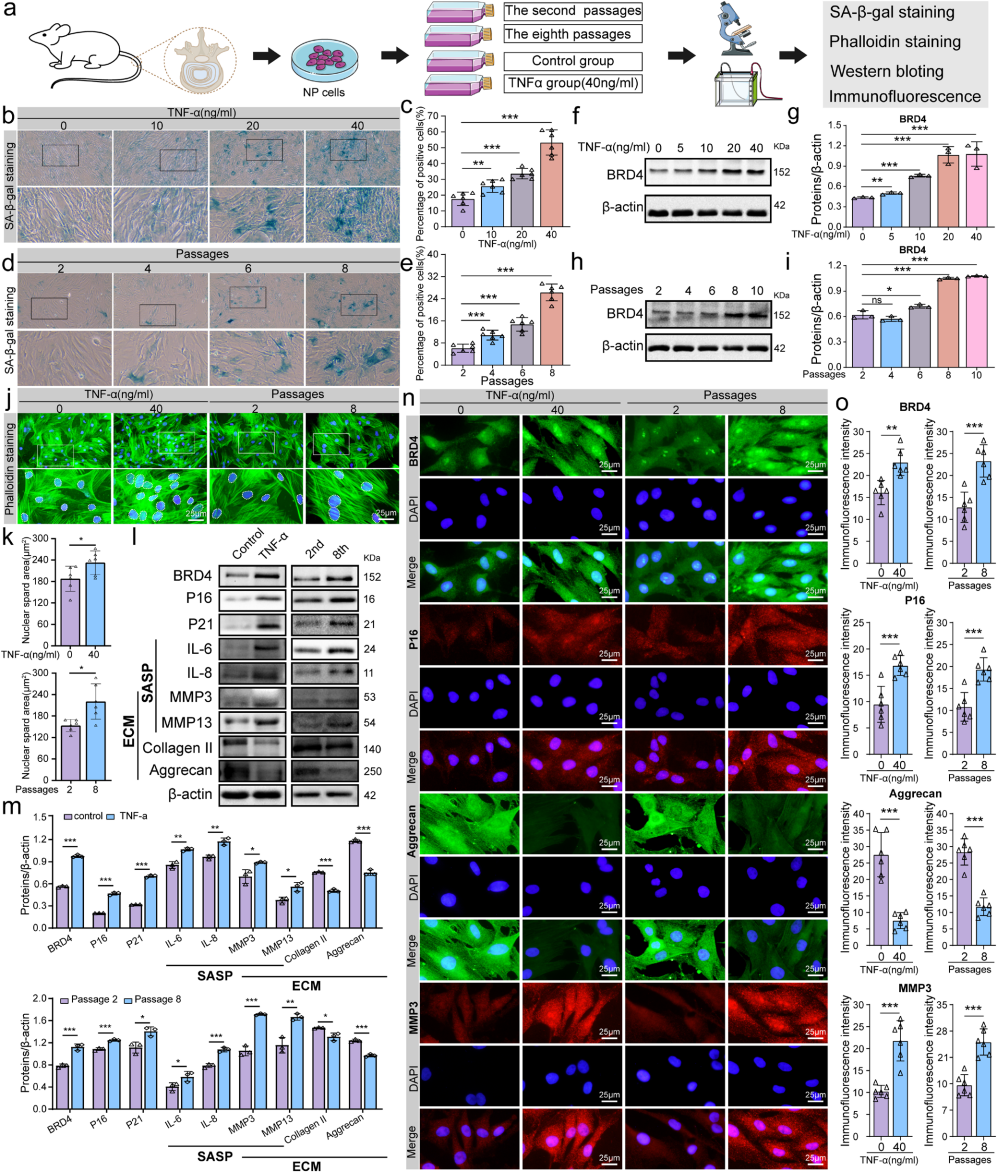
Elevated BRD4 levels in senescent NP cells. (a) Flowchart illustrating the process of establishing the cell senescence model. (b, c) Representative images and quantitative analysis of SA‐β‐gal staining to evaluate senescence levels in NP cells exposed to varying concentrations of TNF‐α. (d, e) Representative images and quantitative analysis of SA‐β‐gal staining to assess the level of senescence in NP cells with increasing passage numbers. Enhanced blue staining in SA‐β‐gal indicates higher enzymatic activity and increased cellular senescence. (f, g) Representative Western blot images and quantitative analysis of BRD4 protein levels in NP cells following treatment with different concentrations of TNF‐α. (h, i) Representative Western blot images and analysis of BRD4 protein expression in NP cells at different passage numbers. (j, k) Representative images and quantitative analysis of phalloidin staining in NP cells across different groups. Larger nuclear volume and more pronounced irregularities signify greater cellular senescence. (l, m) Representative Western blot images and quantitative analysis of P16, P21, IL‐6, IL‐8, MMP3, MMP13, collagen II, and aggrecan expression levels in NP cells of different groups. (n, o) Representative images and quantitative analysis of immunofluorescence staining for BRD4, P16, aggrecan, and MMP3 in the two cell senescence models. Data are presented as mean ± SD. *p < 0.05, **p < 0.01, ***p < 0.001, ns, not statistically significant.

Recent studies have extensively used the TNF‐α‐induced NP cell degeneration model to explore mechanisms related to IDD. Research has established a significant correlation between IDD and NP cell senescence (Chen et al. 2023). Replicative senescence, a primary form of senescence occurring during cell growth, has been identified as a crucial mechanism in human aging (Bitencourt et al. 2024; Iordache et al. 2024). In this study, we developed two cellular senescence models: a TNF‐α‐induced senescence model and a replicative senescence model (Figure 3a). Initial results from SA‐β‐gal staining, following a 48‐h intervention on NP cells with varying TNF‐α concentrations (0, 10, 20, and 40 ng/mL), revealed an increase in cell senescence proportional to the TNF‐α concentration (Figure 3b,c). Furthermore, NP cells from the second, fourth, sixth, and eighth generations were stained for SA‐β‐gal (Figure 3d,e), indicating that senescence increased with the number of cell passages. These results suggest the successful establishment of a senescence model for NP cells. Western blot analysis demonstrated a progressive increase in BRD4 expression with higher concentrations of TNF‐α (Figure 3f,g). Similarly, in the replicative senescence model, BRD4 expression was significantly upregulated as cell passages increased (Figure 3h,i). These findings suggest that BRD4 expression is closely associated with the augmentation of NP cell senescence. Consequently, TNF‐α at 40 ng/mL for 48 h was selected to establish the TNF‐α‐induced cellular senescence model, whereas eighth‐generation cells were selected for the replicative senescence model. Phalloidin staining revealed that TNF‐α treatment at 40 ng/mL significantly increased nuclear volume. In the replicative senescence model (eighth generation), nuclear volume also increased, and typical senescent morphological features appeared (Figure 3j,k). Western blot analysis further confirmed that NP cell senescence led to a marked increase in BRD4, P16, P21, IL‐6, IL‐8, MMP3, and MMP13 expression, accompanied by a decrease in collagen II and aggrecan expression (Figure 3l,m). These findings were corroborated by immunofluorescence results (Figure 3n,o). In summary, our investigation of two cellular senescence models confirmed an increase in BRD4 expression as NP cells transitioned into senescence, suggesting a potentially crucial role for BRD4 in this process.

**FIGURE 4:**
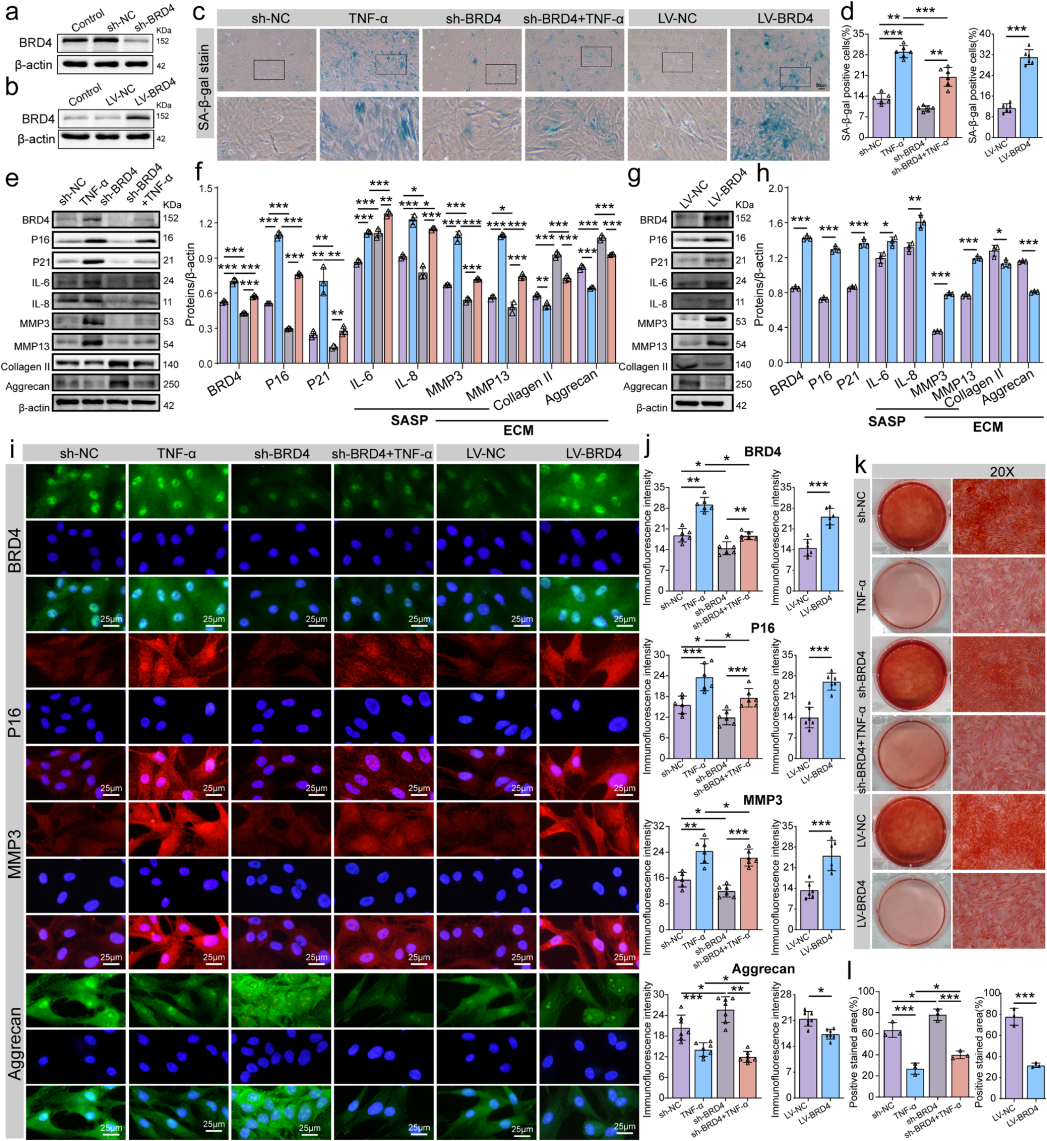
BRD4 mediates NP cell senescence and ECM metabolism. (a) Western blot confirming the knockdown of BRD4 in NP cells post‐transfection with BRD4 shRNA lentivirus. (b) Western blot showing BRD4 overexpression in NP cells following transfection with BRD4 gene‐sequence lentivirus (LV‐BRD4). (c, d) Representative images and quantitative analysis of SA‐β‐gal staining to evaluate the extent of senescence across different groups. The intensity of blue staining serves as an indicator of elevated SA‐β‐gal activity, suggesting a higher degree of cellular senescence. (e–h) Representative Western blot images and quantitative analysis of P16, P21, IL‐6, IL‐8, MMP3, MMP13, collagen II, and aggrecan expression levels in NP cells following BRD4 knockdown and overexpression. (i, j) Representative images and quantitative analysis of immunofluorescence staining for BRD4, P16, aggrecan, and MMP3 across different groups. (k, l) Representative images and quantitative analysis of Safranin O staining in various groups. Data are presented as mean ± SD. *p < 0.05, **p < 0.01, ***p < 0.001.

To explore further the role of altered BRD4 expression in NP cells, we constructed NP cells with both BRD4 knockdown and overexpression. Validation of BRD4 knockdown and overexpression is shown in Figure 4a,b. SA‐β‐gal staining revealed that TNF‐α treatment accelerated NP cell senescence, while BRD4 knockdown significantly reduced the proportion of senescent cells. Conversely, BRD4 overexpression led to a substantial increase in cell senescence (Figure 4c,d). Western blot analysis showed that, under TNF‐α treatment, P16, P21, IL‐6, IL‐8, MMP3, and MMP13 levels increased, whereas collagen II and aggrecan expression decreased. BRD4 knockdown significantly reduced P16, P21, and SASP marker expression, and shifted ECM metabolism from catabolism to anabolism—as evidenced by reduced MMP3 and MMP13 expression and increased collagen II and aggrecan levels. Conversely, BRD4 overexpression accelerated NP cell senescence, increased ECM catabolism, and decreased anabolism (Figure 4e–h). Immunofluorescence results were consistent with the Western blot findings (Figure 4i,j). Saffron O staining further illustrated the enhancement of ECM synthesis following BRD4 downregulation (Figure 4k,l).

### 2.4 MAP2K7 Is a Downstream Target of BRD4

**FIGURE 5:**
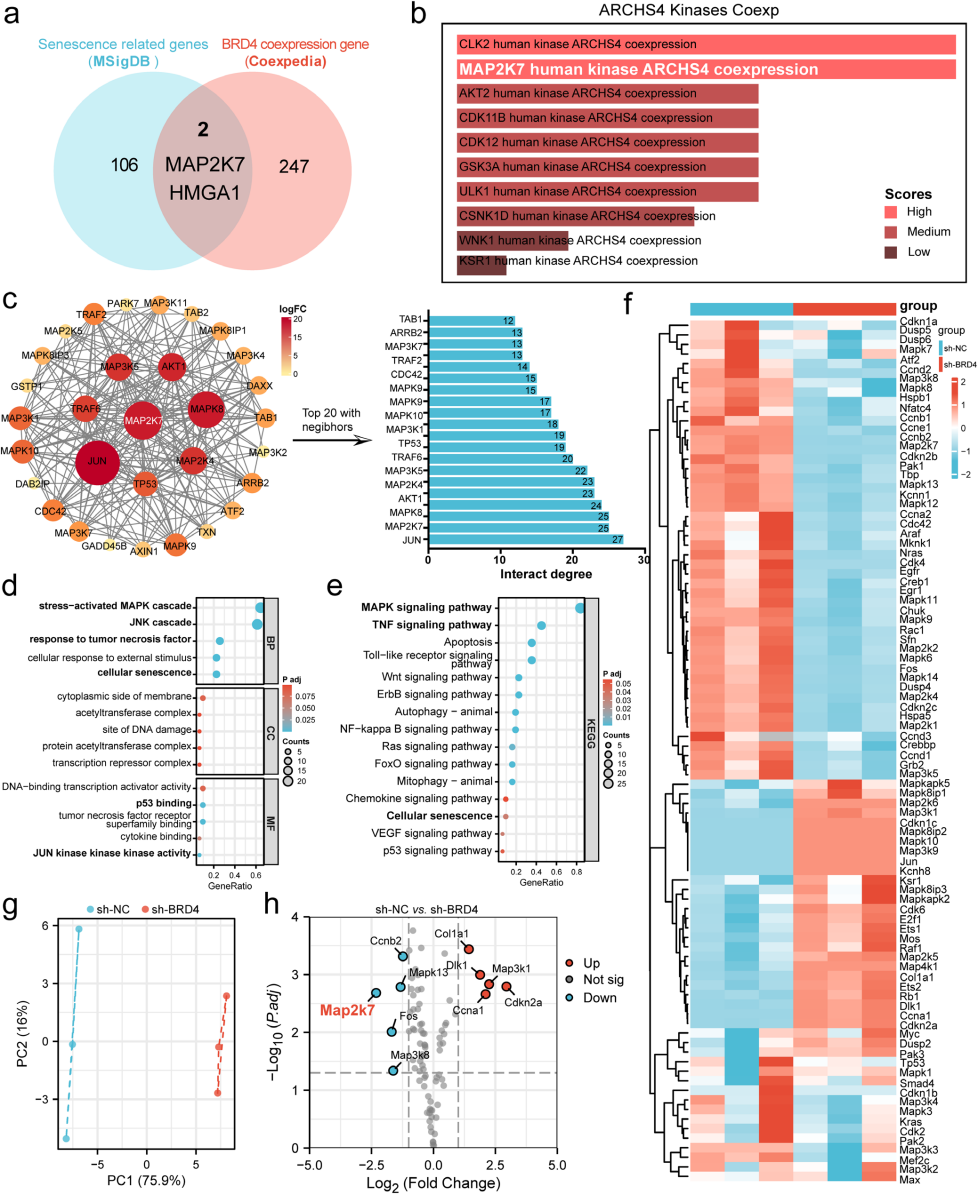
MAP2K7 is a downstream target of BRD4. (a) Venn diagram depicting the intersection of BRD4 co‐expressed genes from the Coexpedia database and senescence‐associated genes from the MSigDB database, identifying MAP2K7 and HMGA1 as genes that mediate cellular senescence and are co‐expressed with BRD4. (b) BRD4 co‐expressed genes identified using the Enrichr database. (c) Co‐expression gene network of BRD4 constructed using the STRING database. (d, e) GO and KEGG pathway analysis of BRD4 co‐expressed genes, performed using the STRING database. (f) Heatmap of PCR Array results indicating changes in MAPK signaling pathway molecules following BRD4 downregulation. Red represents upregulated genes, and blue represents downregulated genes. (g) PCA analysis of PCR Array data. The PCA scatter plot shows each sample as a dot, with high proportions of PC1 and PC2 demonstrating distinct group differences. (h) Volcano plot highlighting upregulated and downregulated genes in the MAPK signaling pathway after BRD4 knockdown. Red dots represent upregulated genes, blue dots represent downregulated genes, and gray dots represent genes with no significant change.

To investigate further the downstream mechanisms by which BRD4 mediates NP cell senescence, we conducted database mining and bioinformatics analyses. First, we obtained BRD4 co‐expressed genes from the Coexpedia database and a list of senescence‐related genes from the MSigDB database. The intersection of these two datasets identified MAP2K7 and HMGA1 as genes co‐expressed with BRD4 and involved in cell senescence (Figure 5a). Subsequently, analysis of the ARCHS4 page from the Enrichr database further confirmed MAP2K7 as a BRD4 co‐expressed gene (Figure 5b). Thus, MAP2K7 was identified as a gene that both co‐expresses with BRD4 and shares mutual regulatory functions. To explore the biological roles mediated by MAP2K7, we constructed a co‐expression network for the MAP2K7 gene using the STRING database (Figure 5c). GO and KEGG enrichment analyses of MAP2K7 co‐expressed genes revealed a strong association with cellular senescence (Figure 5d,e). Previous studies have shown that MAP2K7 is a critical component of the MAPK signaling pathway (Openshaw et al. 2019; Zhang, Shao, et al. 2022). To determine whether MAP2K7 acts as a downstream effector of BRD4, we used a Polymerase Chain Reaction (PCR) Array kit to assess changes in MAPK signaling pathway molecules following BRD4 knockdown. The expression changes in MAPK signaling molecules after BRD4 knockdown were visualized via a heatmap (Figure 5f). Principal component analysis (PCA) of the PCR Array data demonstrated significant differences between groups (Figure 5g). Differentially expressed genes (DEGs) were identified with a cutoff of |logFC| ≥ 1 and *p* < 0.05, yielding 10 DEGs—5 upregulated and 5 downregulated—with MAP2K7 being the most significantly downregulated gene (Figure 5h). Taken together, our analysis confirms that MAP2K7 is a downstream regulatory molecule of BRD4. Therefore, we speculate that MAP2K7 plays a crucial role in the process of NP cell senescence.

### 2.5 MAP2K7 Mediates NP Cell Senescence and ECM Metabolism

**FIGURE 6:**
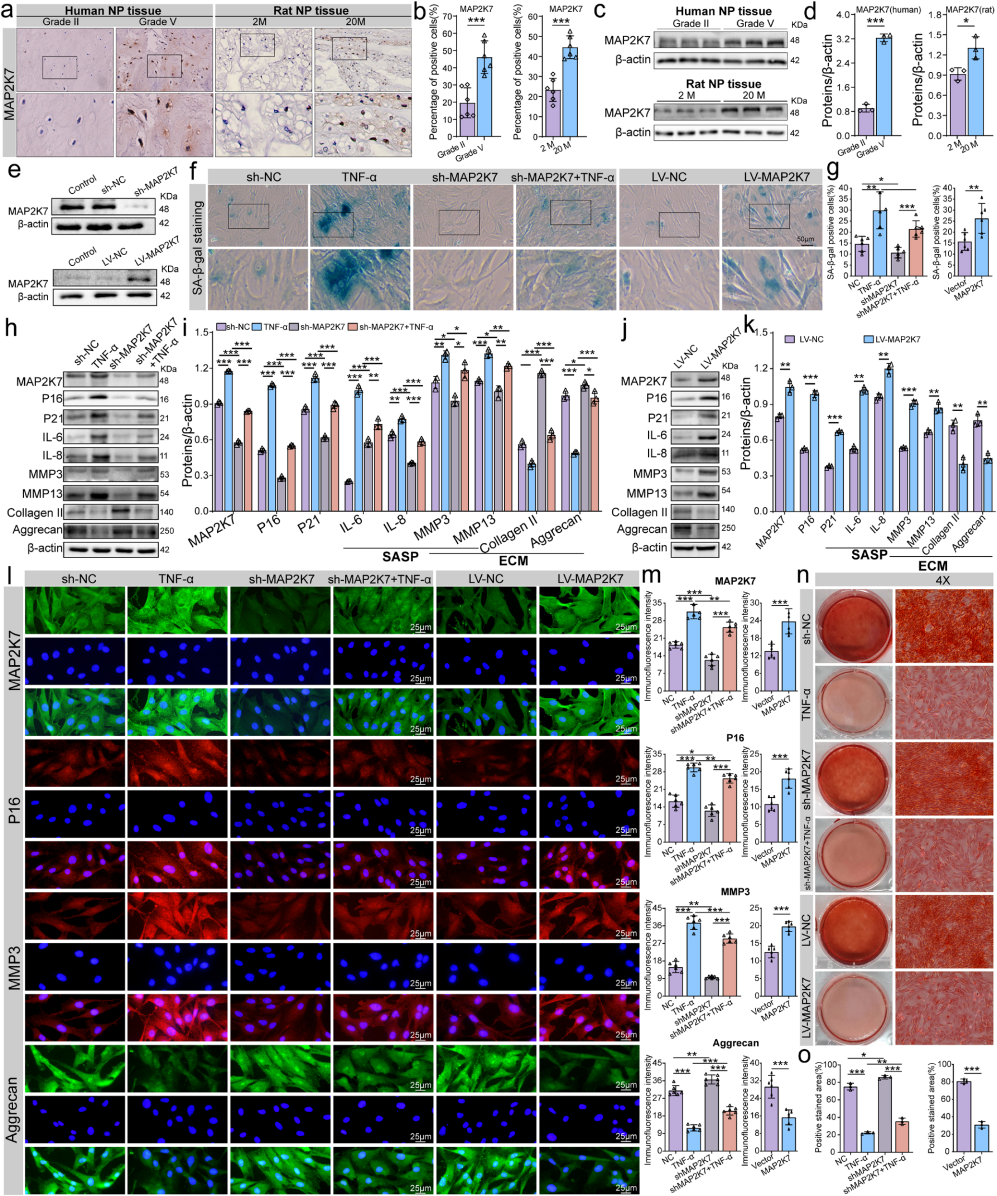
MAP2K7 mediates NP cell senescence and ECM metabolism. (a, b) Representative immunohistochemical images and quantitative analysis of MAP2K7 expression in NP tissues from Grade II and Grade V patients with IDD, as well as from young (2 M) and aged (20 M) rats. (c, d) Representative Western blot bands and quantitative analysis of MAP2K7 expression in NP tissues from Grade II and Grade V patients, and from young (2 M) and aged (20 M) rats. (e) Western blot validation showing MAP2K7 knockdown in NP cells after transfection with MAP2K7 shRNA lentivirus and overexpression after transfection with MAP2K7 gene‐sequence lentivirus (LV‐MAP2K7). (f, g) Representative images and quantitative analysis of SA‐β‐gal staining used to evaluate the level of senescence across different intervention groups. Increased blue staining reflects higher SA‐β‐gal activity, indicating greater cellular senescence. (h–k) Representative Western blot bands and quantitative analysis of P16, P21, IL‐6, IL‐8, MMP3, collagen II, and aggrecan expression levels following MAP2K7 knockdown and overexpression in NP cells. (l, m) Representative images and quantitative analysis of immunofluorescence staining for MAP2K7, P16, aggrecan, and MMP3 in NP cells from different groups. (n, o) Representative images and quantitative analysis of Safranin O staining in different groups. Data are expressed as mean ± SD. *p < 0.05, **p < 0.01, ***p < 0.001.

First, we examined MAP2K7 expression changes in NP tissues from patients with IDD and aged SD rats. Immunohistochemistry revealed a significant increase in the positive expression rate of MAP2K7 in Grade V human NP tissues. Similarly, aged rats (20 M) exhibited a marked increase in the positive expression rate of MAP2K7 in NP tissues (Figure 6a,b). These findings were corroborated by Western blot analysis (Figure 6c,d). To investigate the role of altered MAP2K7 expression in NP cells, we generated NP cells with both MAP2K7 knockdown and overexpression. The validation of MAP2K7 knockdown and overexpression is shown in Figure 6e. SA‐β‐gal staining revealed that, under TNF‐α treatment, NP cell senescence was accelerated, and MAP2K7 knockdown significantly reduced the proportion of senescent NP cells. Conversely, MAP2K7 overexpression significantly increased the level of cell senescence (Figure 6f,g). Western blot analysis indicated that TNF‐α treatment led to elevated expression levels of P16 and P21, along with increased levels of SASP markers (IL‐6, IL‐8, MMP3, and MMP13). Additionally, the expression levels of ECM markers, such as collagen II and aggrecan, decreased, whereas MMP3 and MMP13 levels increased. After MAP2K7 knockdown, the expression of P16, P21, and SASP markers was significantly reduced. ECM metabolism shifted from catabolism to anabolism, demonstrated by a reduction in MMP3 and MMP13 levels and an increase in collagen II and aggrecan levels. Conversely, MAP2K7 overexpression had the opposite effect, promoting NP cell senescence and enhancing ECM catabolism while reducing anabolism (Figure 6h,k). Immunofluorescence findings aligned with the Western blot results (Figure 6l,m). Moreover, Saffron O staining demonstrated that BRD4 downregulation accelerated ECM synthesis and reduced ECM degradation (Figure 6n,o). These results suggest that MAP2K7 upregulation significantly enhances NP cell senescence and promotes ECM catabolism while diminishing anabolism. Conversely, MAP2K7 downregulation exhibits the opposite effects.

### 2.6 BRD4 and MAP2K7 Co‐Regulate PGF Expression

**FIGURE 7:**
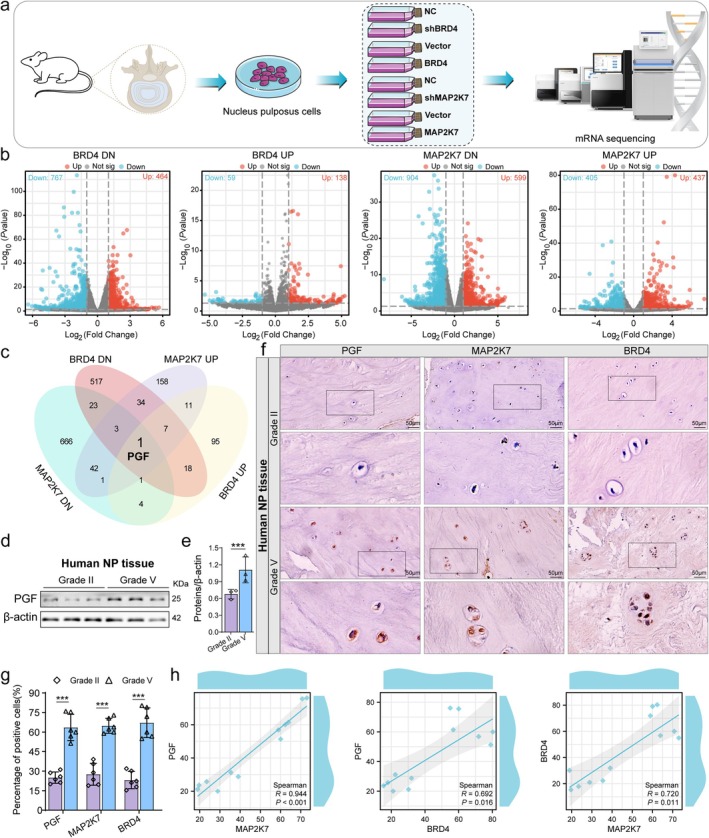
BRD4 and MAP2K7 co‐regulate PGF expression. (a) Schematic diagram of transcriptome sequencing performed after BRD4 and MAP2K7 knockdown and overexpression. (b) Volcano plots illustrating DEGs following BRD4 and MAP2K7 knockdown and overexpression, with |logFC| ≥ 1 and *p* < 0.05 as the threshold. Red dots represent upregulated genes, whereas blue dots indicate downregulated genes. (c) Venn diagram showing the overlap of downregulated genes after BRD4 knockdown, upregulated genes after BRD4 overexpression, downregulated genes after MAP2K7 knockdown, and upregulated genes after MAP2K7 overexpression. PGF is identified as a downstream gene co‐regulated by both BRD4 and MAP2K7. (d, e) Representative Western blot bands and quantitative analysis of PGF expression in NP tissues from Grade II and Grade V patients with IDD. (f, g) Representative immunohistochemical images and quantitative analysis of PGF, MAP2K7, and BRD4 expression in NP tissues from Grade II and Grade V patients with IDD. (h) Quantitative analysis of immunohistochemical expression levels of PGF, BRD4, and MAP2K7. Data are presented as mean ± SD. ***p < 0.001.

To explore further the downstream mechanisms through which the BRD4/MAP2K7 signaling axis mediates NP cell senescence, we performed transcriptome sequencing analysis following the knockdown and overexpression of BRD4 and MAP2K7, respectively (Figure 7a). DEGs were visualized using volcano plots (Figure 7b). This study aimed to identify downstream genes concurrently regulated by BRD4 and MAP2K7 by examining genes that exhibited downregulation upon BRD4 knockdown, upregulation upon BRD4 overexpression, downregulation following MAP2K7 knockdown, and upregulation following MAP2K7 overexpression. The findings revealed that PGF is a downstream target co‐regulated by both BRD4 and MAP2K7(Figure 7c).

**FIGURE 8:**
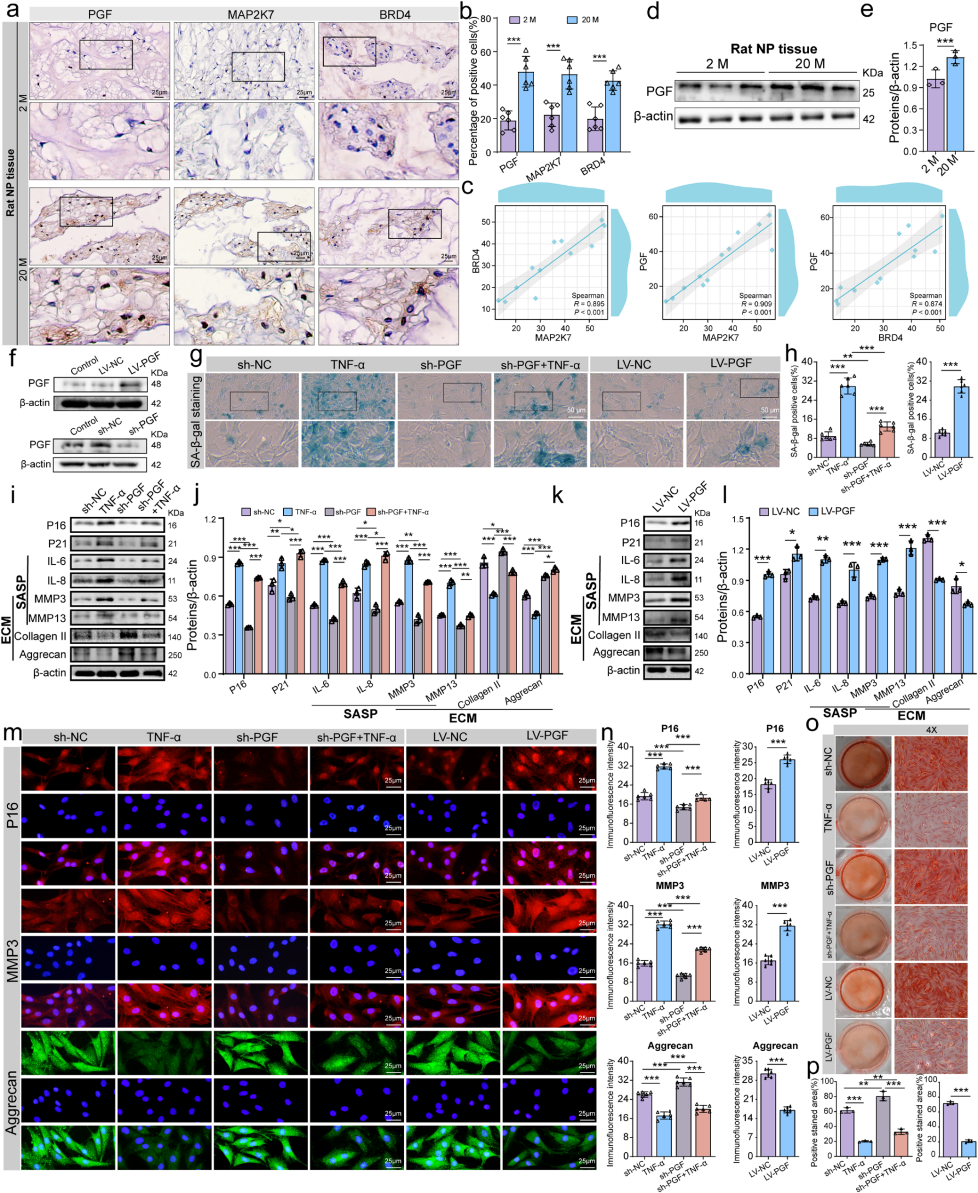
PGF mediates NP cell senescence and ECM metabolism. (a, b) Representative immunohistochemical images and quantitative analysis of PGF expression in NP tissues from young (2 months, 2 M) and aged (20 months, 20 M) rats. (c) Quantitative analysis of immunohistochemical expression levels of PGF, BRD4, and MAP2K7. (d, e) Representative Western blot bands and quantitative analysis of PGF expression in NP tissues from young (2 M) and aged (20 M) rats. (f) Western blot validation of PGF knockdown in NP cells following transfection with PGF shRNA lentivirus, and overexpression after transfection with PGF gene‐sequence lentivirus (LV‐PGF). (g, h) Representative images and quantitative analysis of SA‐β‐gal staining to evaluate the level of senescence in different groups. The intensity of blue staining reflects higher SA‐β‐gal activity, indicating more pronounced cellular senescence. (i–l) Representative Western blot bands and quantitative analysis of P16, P21, IL‐6, IL‐8, collagen II, and aggrecan expression levels after PGF knockdown and overexpression in NP cells. (m, n) Representative images and quantitative analysis of immunofluorescence staining for P16, aggrecan, and MMP3 across different groups. (o, p) Representative images and quantitative analysis of Safranin O staining in different groups. Data are presented as mean ± SD. *p < 0.05, **p < 0.01, ***p < 0.001.

Subsequently, we evaluated the differential expression of PGF in NP tissues from patients with IDD. Western blot analysis revealed a significant increase in PGF expression in Grade V NP tissues (Figure 7d,e). Immunohistochemical analysis further demonstrated that, in human NP tissues, the positive expression rates of PGF, MAP2K7, and BRD4 were significantly higher in Grade V compared with Grade II (Figure 7f,g). Correlation analysis revealed a significant positive correlation between PGF and MAP2K7, as well as between MAP2K7 and BRD4, in the NP tissues of patients with IDD (Figure 7h). We also assessed the differential expression of PGF in the NP tissues of aged SD rats, which showed results consistent with the expression pattern of PGF in human NP tissues (Figure 8a–e).

### 2.7 PGF Mediates NP Cell Senescence and ECM Metabolism

To investigate the role of altered PGF expression in NP cells, we generated NP cells with both PGF knockdown and overexpression. Validation of PGF knockdown and overexpression is shown in Figure 8f. SA‐β‐gal staining indicated that the knockdown of PGF significantly reduced the proportion of senescent NP cells, whereas the overexpression of PGF markedly increased cell senescence levels (Figure 8g,h). Western blot analysis demonstrated that, following TNF‐α treatment, the expression levels of P16, P21, and SASP markers were significantly elevated. Concurrently, ECM markers such as collagen II and aggrecan were reduced, whereas MMP3 and MMP13 levels increased. After PGF knockdown under these conditions, P16, P21, and SASP markers were notably decreased, with a shift in ECM metabolism from catabolic to anabolic processes. Conversely, PGF overexpression produced the opposite effects, accelerating NP cell senescence and enhancing ECM catabolism while reducing anabolic activity (Figure 8i–l). Immunofluorescence findings were consistent with the Western blot results (Figure 8m,n). Saffron O staining further demonstrated that PGF downregulation accelerated ECM synthesis and decreased degradation (Figure 8o,p). These findings suggest that PGF downregulation significantly reduces NP cell senescence, inhibits ECM catabolism, and promotes anabolic processes.

### 2.8 BRD4/MAP2K7/PGF Signaling Axis Mediates NP Cell Senescence and ECM Metabolism

**FIGURE 9:**
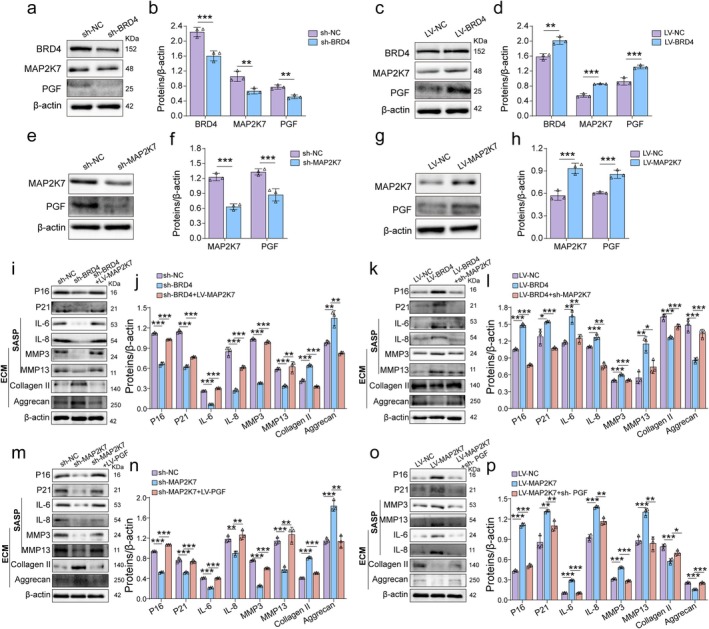
BRD4/MAP2K7/PGF signaling axis mediates NP cell senescence and ECM metabolism. (a–d) Representative Western blot bands and quantitative analysis of BRD4, MAP2K7, and PGF expression levels after BRD4 knockdown and overexpression in NP cells. (e–h) Representative Western blot bands and quantitative analysis of MAP2K7 and PGF expression levels after MAP2K7 knockdown and overexpression in NP cells. (i, j) Representative Western blot bands and quantitative analysis of P16, P21, IL‐6, IL‐8, MMP3, MMP13, collagen II, and aggrecan expression levels after MAP2K7 overexpression in BRD4 knockdown NP cells. (k, l) Representative Western blot bands and quantitative analysis of P16, P21, IL‐6, IL‐8, MMP3, MMP13, collagen II, and aggrecan expression levels in NP cells after MAP2K7 knockdown in BRD4 overexpressed cells. (m, n) Representative Western blot bands and quantitative analysis of P16, P21, IL‐6, IL‐8, MMP3, MMP13, collagen II, and aggrecan expression levels after PGF overexpression in MAP2K7 knockdown cells. (o, p) Representative Western blot bands and quantitative analysis of P16, P21, IL‐6, IL‐8, MMP3, MMP13, collagen II, and aggrecan expression levels after PGF knockdown in MAP2K7 overexpressed cells across different groups. Data are presented as mean ± SD. *p < 0.05, **p < 0.01, ***p < 0.001.

To confirm that the BRD4/MAP2K7/PGF axis mediates NP cell senescence and ECM metabolism, we conducted functional rescue experiments. Initially, we performed knockdown and overexpression of BRD4 to clarify the expression of MAP2K7 and PGF, and subsequently assessed the expression of PGF following the knockdown and overexpression of MAP2K7. Western blot analysis indicated that the expression levels of MAP2K7 and PGF decreased after BRD4 knockdown, while they increased following BRD4 overexpression (Figure 9a–d). After MAP2K7 knockdown, the expression of PGF was reduced, whereas PGF expression increased following MAP2K7 overexpression (Figure 9e–h). Subsequently, we knocked down BRD4 while overexpressing MAP2K7 to assess cell senescence levels and ECM metabolism. Western blot analysis indicated that with BRD4 knockdown and simultaneous overexpression of MAP2K7, the expression levels of senescence markers P16 and P21, as well as SASP markers, decreased, while ECM catabolism increased and anabolism decreased (Figure 9i,j). Conversely, with BRD4 overexpression and simultaneous knockdown of MAP2K7, the senescence markers in NP cells significantly decreased, and ECM anabolism was enhanced while catabolism was reduced (Figure 9k,l). Furthermore, PGF overexpression following MAP2K7 knockdown resulted in decreased expression of senescence markers P16 and P21 and SASP markers, with enhanced ECM catabolism and reduced anabolism (Figure 9m,n). When MAP2K7 was overexpressed and PGF was simultaneously knocked down, the senescence markers in NP cells significantly decreased, ECM anabolism was enhanced, and catabolism was reduced (Figure 9o,p). Collectively, these results confirm that the BRD4/MAP2K7/PGF signaling axis mediates NP cell senescence and ECM metabolism.

### 2.9 BRD4/MAP2K7/PGF Signaling Axis Mediates NP Cell Senescence and ECM Metabolism in Needle Puncture‐Induced IDD Rat

**FIGURE 10:**
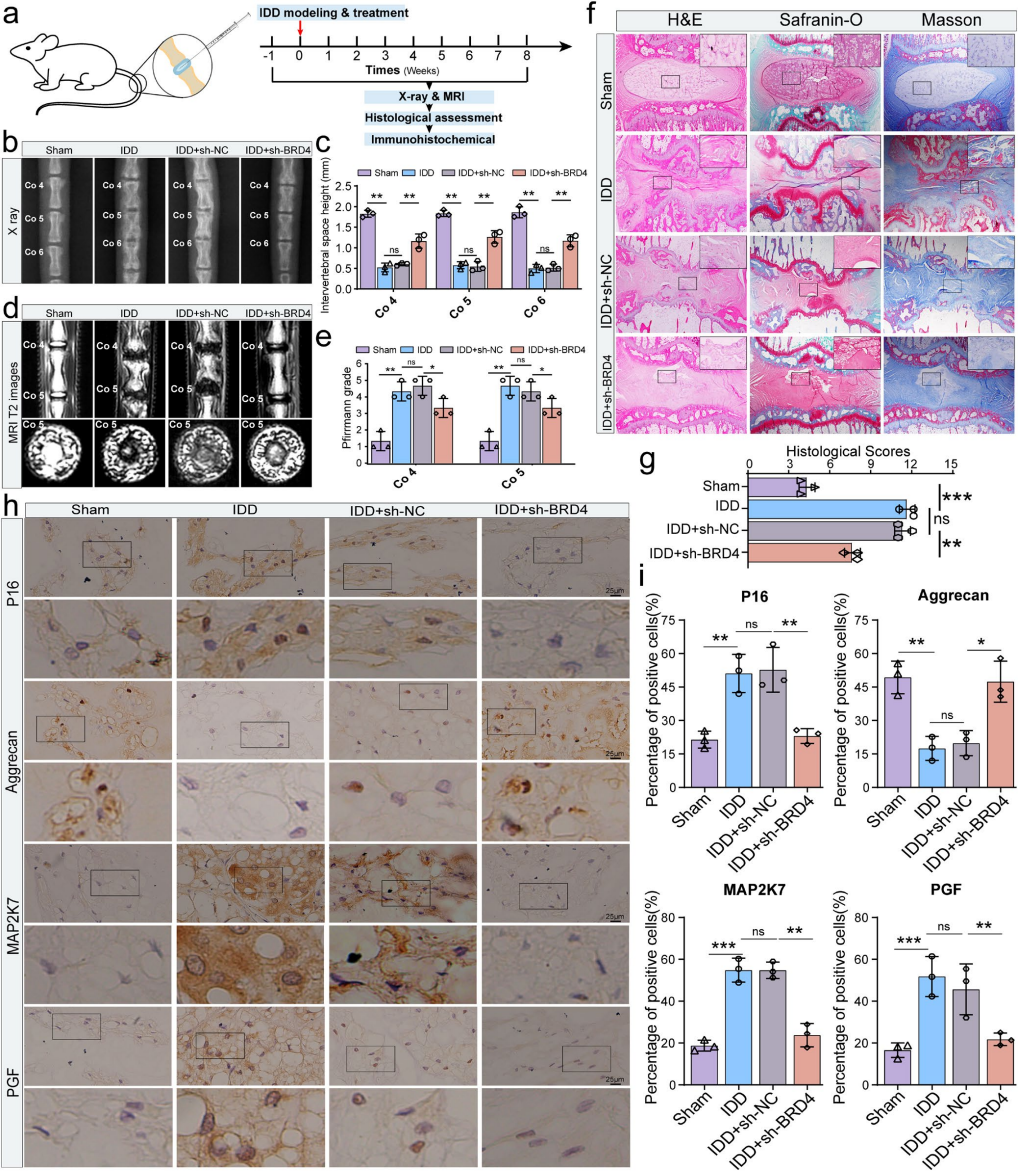
BRD4/MAP2K7/PGF signaling axis mediates NP cell senescence and ECM metabolism in needle puncture‐induced IDD rats. (a) Schematic diagram showing the establishment and evaluation of a rat IDD model induced by needle puncture. (b, c) Representative X‐ray images and quantitative analysis of disk height in different groups. (d, e) Representative T2‐weighted MRI of different groups and Pfirrmann grade quantitative analysis. (f, g) Representative images of H&E staining, Safranin O‐Fast Green staining, and Masson's trichrome staining in different groups, accompanied by quantitative analysis of histological scores from H&E staining. (h, i) Representative images and quantitative analysis of senescence marker P16, ECM synthesis marker Aggrecan, and BRD4 downstream molecules MAP2K7 and PGF protein expression in NP tissues from rat caudal vertebrae across different groups. Data are presented as mean ± SD. *p < 0.05, **p < 0.01, ***p < 0.001.

To verify further the key role of BRD4 in IDD, we established a needle puncture‐induced rat IDD model for in vivo validation (Figure 10a). X‐ray results showed that, compared with the model group, disk height partially recovered after BRD4 knockdown (Figure 10b,c). Following lentivirus‐mediated sh‐BRD4 treatment, T2‐weighted MRI indicated a recovery of the NP signal, a reduction in the Pfirrmann grade, and partial restoration of the NP tissue area (Figure 10d,e). H&E staining demonstrated an increase in the number of NP cells, fewer annulus fibrosus ruptures, and the presence of a gel‐like ECM in the NP after sh‐BRD4 treatment. Saffron O‐Fast Green and Masson staining further revealed an increase in collagen and proteoglycan synthesis following BRD4 knockdown (Figure 10f,g).

Next, we evaluated the relationship between BRD4 knockdown and NP cell senescence and ECM metabolism. Immunohistochemistry showed that after sh‐BRD4 treatment, P16 expression decreased, whereas aggrecan expression increased (Figure 10h,i). These findings indicate that BRD4 knockdown has a significant protective effect against needle puncture‐induced IDD in rats, primarily by reducing NP cell senescence and ECM degradation. Additionally, we examined the expression changes in downstream molecules MAP2K7 and PGF following BRD4 knockdown. Immunohistochemistry results revealed that the expression of MAP2K7 and PGF also changed in correlation with BRD4, indicating that the BRD4/MAP2K7/PGF signaling axis mediates the progression of IDD in vivo (Figure 10h,i).

## 3 Discussion

As the population ages, LBP has become a leading cause of disability and a major reason for medical visits worldwide. IDD is the primary cause of LBP, but its underlying mechanisms are not well understood. Accelerated senescence of disk cells due to abnormal factors is thought to trigger IDD. The hallmark of cellular senescence is the increase in SA‐β‐gal‐positive cells and the upregulation of cyclin‐dependent kinase inhibitors, such as P16 and P21. Additionally, senescent cells can secrete SASP, which typically involves a range of pro‐inflammatory factors (e.g., IL‐1α, IL‐1β, IL‐6, and IL‐8), growth factors (e.g., HGF, TGF‐β, and GM‐CSF), chemokines (e.g., CXCL‐1/3 and CXCL‐10), and matrix remodeling enzymes such as MMPs, including MMP3, MMP9, and MMP13. These cytokines influence the cellular microenvironment, accelerating the senescence of surrounding cells (López‐Otín et al. 2023, 2013; Hernandez‐Segura et al. 2018).

BRD4, a member of the BET protein family, plays a crucial role in regulating gene transcription processes involved in cellular senescence, autophagy, inflammation, apoptosis, and extracellular matrix (ECM) metabolism (Wei et al. 2024; Zhang and Roeder 2025; Wang et al. 2019; Zhang, Chen, et al. 2022). Recent studies have increasingly focused on the role of BRD4 in the skeletal system, particularly with regard to IDD. Wang et al. (Wang et al. 2019; Openshaw et al. 2019) demonstrated that BRD4 expression is elevated in diabetic IDD and that BRD4 knockdown ameliorates diabetic IDD by suppressing the expression of matrix metalloproteinase MMP13 through regulation of the MAPK and NF‐κB signaling pathways, as well as the autophagy process. Similarly, Hong et al. (Hong et al. 2020) reported that BRD4 knockdown mitigates ECM degradation in NP cells by activating autophagy and inhibiting NLRP3 inflammasome activity via the NF‐κB pathway, thereby exerting an anti‐IDD effect. In our previous studies, we found that BRD4 expression levels are abnormally elevated in NP tissues of patients with IDD and positively correlate with the degree of degeneration, indicating the potential role of BRD4 in IDD (Zhang, Chen, et al. 2022).

In this study, we first constructed a BRD4 co‐expression gene network, followed by GO and KEGG analyses, revealing a close association between BRD4 and the biological process of cellular senescence. Previous research has confirmed that BRD4‐mediated cellular senescence is closely related to IDD, osteoarthritis, atherosclerosis, cancer, and other conditions (Donati et al. 2018; Li et al. 2023; Jiang et al. 2017). Our results align with these previous findings. Subsequently, we established two cellular senescence models to investigate the expression changes and mechanisms of BRD4. In recent years, TNF‐α‐induced NP cell degeneration models have been widely used in studies of IDD mechanisms. Li and An (2023) demonstrated that TNF‐α at 20 ng/mL could induce senescence in NP cells. Chen et al. found that TNF‐α at 50 ng/mL, acting on NP cells for 72 h, could establish a stable cellular senescence model. Yang et al. (2022) also reported that TNF‐α at 20 ng/mL, acting on NP cells for 72 h, could create a stable senescence model. Our results indicate that TNF‐α at 40 ng/mL for 48 h can significantly induce senescence in rat NP cells, increasing the proportion of senescent cells to 50%–60% and displaying SASP‐related markers, consistent with previous findings. Previous studies have shown that IDD is closely associated with NP cell senescence, with replicative senescence being one of the main forms of senescence during cell growth (Zhang, Chen, et al. 2022; Silwal et al. 2023). Previous research has demonstrated a significant increase in SA‐β‐gal staining by the eighth generation of NP cells (Zhang, Chen, et al. 2022). In this study, we selected the second, fourth, sixth, and eighth generations of NP cells for SA‐β‐gal staining to assess changes in replicative senescence levels. We observed a significant increase in senescent cells by the eighth generation. Based on these two cellular senescence models, we examined changes in BRD4 expression. Our results showed that BRD4 expression significantly increased with cellular senescence after 48 h of exposure to TNF‐α at 40 ng/mL. In the replicative senescence model, we also detected a significant increase in BRD4 expression once the cells entered replicative senescence. We reliably observed differences in BRD4 expression across both cellular senescence models.

Next, we examined the functional changes in NP cells after knocking down and overexpressing BRD4 using lentiviral transfection. Our results indicated that BRD4 expression increased under TNF‐α stimulation, correlating with the onset of cellular senescence. When BRD4 was knocked down, there was a significant reduction in NP cell senescence, along with decreased ECM catabolism and increased anabolic activity. Conversely, increased BRD4 expression in normal NP cells led to a marked elevation in cellular senescence, enhanced ECM catabolism, and reduced anabolic activity. Previous studies have shown that in an LPS‐induced macrophage senescence model, BRD4 expression increases, and BRD4 downregulation significantly reduces macrophage senescence (Wang et al. 2020). Other studies have confirmed that the inflammatory mediator LPS induces the redistribution of BRD4 on chromosomes via activation of the NF‐κB signaling pathway, enhancing the expression of SASP‐related genes and promoting the senescent phenotype of macrophages through a paracrine mechanism (Shanley et al. 2009). Thompson et al. (2019) found that BRD4 is a key transcriptional activator of SASP in mouse and human pancreatic cells, and that BET inhibitors provide protection against diabetes by suppressing BRD4 expression and reducing SASP‐related molecule expression. Tasdemir et al. (2016) demonstrated that BRD4 downregulation reduces senescence in human fibroblasts by inhibiting the expression of SASP‐related molecules. These findings align with our results.

To investigate the downstream mechanisms by which BRD4 mediates NP cell senescence, we conducted database mining and bioinformatics analysis, preliminarily identifying MAP2K7 as a BRD4 co‐expressed gene with mutual regulatory interactions. We analyzed the protein–protein interaction network of the MAP2K7 protein and performed GO and KEGG enrichment analyses, which indicated that MAP2K7 is involved in the biological process of cellular senescence. Considering that MAP2K7 is an important component of the MAPK signaling pathway, we further explored whether MAP2K7 acts as a downstream molecule of BRD4. Using a medium‐throughput PCR array, we examined changes in MAPK signaling pathway molecules following BRD4 downregulation. The results showed that MAP2K7 was significantly downregulated after BRD4 knockdown, further confirming our hypothesis that MAP2K7 is a downstream molecule of BRD4 and mediates NP cell senescence.

To explore the role of reduced MAP2K7 expression in NP cells, we knocked down and overexpressed MAP2K7 to observe its functional effects. The results demonstrated that MAP2K7 expression increased under TNF‐α stimulation, and MAP2K7 knockdown significantly reduced NP cell senescence, weakened ECM catabolism, and enhanced anabolic activity. Conversely, when MAP2K7 expression was increased, cellular senescence was markedly elevated, along with enhanced ECM catabolism and reduced anabolic activity. Additionally, we confirmed the differential expression of MAP2K7 in clinical NP tissue and aged rat NP tissue. MAP2K7, also known as MKK7 or MEK7, plays a crucial role in the development of various age‐related diseases (Li et al. 2020; Lu et al. 2022; Zhang, Lu, et al. 2023). Li et al. (2020) demonstrated that the ASK1/MKK7/JNK signaling pathway plays a crucial role in hypothalamic neuron cell senescence. Zhang, Shao, et al. (2022) revealed that excessive mechanical loading promotes chondrocyte senescence through activation of the MKK7/JNK signaling pathway, thereby accelerating the progression of osteoarthritis.

To investigate the downstream effectors of BRD4/MAP2K7 signaling in NP cell senescence, we manipulated BRD4 and MAP2K7 expression and conducted transcriptome sequencing, identifying PGF as a regulated gene. We then performed PGF knockdown and overexpression experiments in NP cells to analyze its role in IDD. Our findings revealed that PGF expression increases as NP cells become senescent. Reducing PGF levels led to decreased NP cell senescence, reduced ECM breakdown, and enhanced anabolic activity. Conversely, increasing PGF expression accelerated cell senescence, heightened ECM breakdown, and decreased anabolic activity. We also observed varying PGF levels in clinical and aged rat NP tissues. PGF, also known as PIGF or PLGF, is widely implicated in the development of age‐related diseases such as cancer, diabetes, and retinal disorders (Förger et al. 2022; Hookham et al. 2016; Selvaraj et al. 2003; Shimizu et al. 2010; Zhang et al. 2022). For instance, PGF promotes lung cancer metastasis by enhancing MMP9 expression (Zhang et al. 2015). Lazzara et al. (2019) demonstrated that inhibiting the PGF/ERK pathway significantly alleviates high glucose‐induced retinal inflammation, whereas Kang et al. (2018) reported that PGF mediates diet‐induced metabolic disorders in obese mice by regulating inflammatory responses.

Finally, we validated the mutual regulatory relationships between BRD4, MAP2K7, and PGF. Knocking down BRD4 reduced NP cell senescence, decreased ECM catabolism, and enhanced anabolic activity. However, overexpressing MAP2K7 reversed these benefits. Conversely, overexpressing BRD4 increased NP cell senescence, enhanced ECM catabolism, and reduced anabolic activity, but these effects were blocked when MAP2K7 was simultaneously knocked down. Similarly, MAP2K7 knockdown reduced cell senescence, decreased ECM breakdown, and boosted anabolic activity, but overexpression of PGF negated these benefits. Conversely, MAP2K7 overexpression led to increased cell senescence, heightened ECM breakdown, and reduced anabolic activity, while PGF knockdown alongside MAP2K7 overexpression mitigated these effects. These results confirm the interplay between BRD4, MAP2K7, and PGF, showing that the BRD4/MAP2K7/PGF signaling pathway regulates NP cell senescence and ECM metabolism.

Nonetheless, several aspects of our study warrant further refinement. Firstly, although the age‐related models in rats and the acupuncture‐induced models partially replicate the physiological and pathological changes associated with IDD, it is crucial to acknowledge that rodent intervertebral disks differ markedly from those of larger mammals in terms of function and biomechanical properties. Future research should incorporate larger animal models, such as monkeys or goats, which more accurately mimic the biomechanical characteristics of the human spine, to more thoroughly explore the therapeutic effects of BRD4 in IDD and the associated in vivo mechanisms. Secondly, although the rat NP cells employed in this study are well characterized, stable, and readily available, species‐specific functional differences between rat and human NP cells may constrain the applicability of our findings to human physiology. Future investigations should prioritize the extraction of primary human NP cells for further validation.

In conclusion, our study underscores the crucial role of the BRD4/MAP2K7/PGF signaling axis in regulating NP cell senescence and ECM metabolism. This research provides novel insights into the molecular regulatory mechanisms governing NP cell aging. The identification of the BRD4/MAP2K7/PGF signaling axis substantially enriches and advances the theoretical framework for understanding the molecular regulation of IDD, laying a crucial foundation for future in‐depth investigations. Targeting the excessive activation of components within this signaling axis could present promising therapeutic strategies for mitigating IDD.

## 4 Materials and Methods

### 4.1 Ethical Approval

All animal experiments adhered to the ARRIVE guidelines and were reviewed and approved by the Animal Care and Use Committee at the Second Hospital of Lanzhou University (approval no.: D2022‐081).

Human sample experiments conformed to the Declaration of Helsinki and were approved by the Ethical Review Committee of the Second Hospital of Lanzhou University (approval no. 2022A‐138). Written informed consent was obtained from all participants at the time of enrollment.

### 4.2 BRD4 Co‐Expression Gene Construction and Bioinformatics Analysis

A list of BRD4 co‐expressed genes was retrieved from the STRING database (version 11.0, http://www.string‐db.org) and visualized using Cytoscape software (version 3.7.1). GO function enrichment analysis—including cellular component, biological process, and molecular function—as well as KEGG pathway enrichment analysis was conducted. Enrichment results were visualized using the R package enrichplot, with an adjusted *p* value threshold of < 0.05 set for significance.

### 4.3 BRD4 Downstream Gene Prediction

Co‐expressed genes for BRD4 were obtained from the Coexpedia database (www.coexpedia.org), and senescence‐related genes were sourced from the MSigDB database (https://www.gsea‐msigdb.org/gsea/msigdb/). Additional BRD4 co‐expressed genes were identified using the ARCHS4 analysis tool within the Enrichr database (https://maayanlab.cloud/Enrichr/). Intersection analysis, combining data from Coexpedia, MSigDB, and Enrichr, was conducted to identify the co‐expressed genes of BRD4 involved in mediating the senescence phenotype.

### 4.4 Human Samples

Intervertebral disk NP tissue specimens were collected from patients undergoing lumbar spine surgery, in accordance with the Pfirrmann grading system. This study used 24 NP tissue samples from patients who underwent surgery at the Department of Orthopedics, the Second Hospital of Lanzhou University, between June 2020 and December 2023. The samples were categorized into two groups: mild degeneration group (Grade II), which included NP tissue from seven males and five females, aged 22–46 years (mean age: 31.9 years), and severe degeneration group (Grade V), which included NP tissue from eight males and four females, aged 47–69 years (mean age: 57.83 years). The detailed clinical characteristics of the patients are shown in Table S2.

### 4.5 Animals

Healthy male SD rats, aged 4–8 weeks (weighing 200–220 g), were purchased from the Experimental Animal Center of Lanzhou University. SD rats aged 9 months (weighing 300–350 g) and 20 months (weighing 400–450 g) were obtained from Suzhou Xishan Zhongke Laboratory Animal Co. Ltd. All experiments were conducted in a randomized manner, and investigators were blinded to group allocation by coding the rats and samples.

### 4.6 Isolation of Human and Rat NP Cells

Human NP cells were isolated from surgically obtained NP tissue. The tissue was cut into 1‐mm^2^ sections and incubated at 37°C with 5% CO_2_ in DMEM/F‐12 complete medium (supplemented with 10% fetal bovine serum) containing 0.25 mg/mL type II collagenase for 8 h. After digestion, the sediment was separated via centrifugation at 800 rpm for 5 min. Then, the isolated cells were transferred to DMEM/F‐12 complete medium supplemented with 10% fetal bovine serum and cultured, with medium changes every 2–3 days.

For rat NP cell isolation, SD rats (4–6 weeks old) were euthanized by cervical dislocation. The tailbone was excised using tissue scissors, and disk tissues were carefully dissected on a sterile surface. The gelatinous NP tissues were isolated and placed in a dish containing 0.25% collagenase. The tissues were incubated at 37°C for 3–5 h with agitation every 30 min. After digestion, 5 mL of pre‐warmed DMEM/F‐12 complete medium was added, and the mixture was filtered through a 0.75‐μm filter to remove undigested tissue clumps. The filtrate was then evenly spread and transferred to a cell culture flask for incubation at 37°C with 5% CO_2_. Cells were passaged at 80%–90% confluence, typically at a ratio of 1:2 or 1:3.

### 4.7 RNA‐Sequencing and Data Analysis

Total RNA was extracted using TRIzol reagent following the manufacturer's instructions. The purity and concentration of the extracted RNA were measured using a NanoDrop 2000 spectrophotometer (Thermo Fisher Scientific, Waltham, MA, USA), whereas RNA integrity was assessed with a 2100 Bioanalyzer (Agilent Technologies, Santa Clara, CA, USA). RNA‐seq libraries were constructed using the VAHTS Universal V5 RNA‐seq Library Prep Kit, according to the manufacturer's protocol. Sequencing was performed on the Illumina NovaSeq 6000 platform, generating 150‐bp paired‐end reads. Transcriptome sequencing and subsequent analysis were carried out by OE Biotech Co. Ltd. (Shanghai, China).

### 4.8 Immunofluorescence

NP cells were fixed with 4% paraformaldehyde (Beyotime, Shanghai, China) for 15 min, washed with phosphate‐buffered saline, and permeabilized using Triton X‐100 (Servicebio, Wuhan, China) for 25 min. Blocking was performed with 5% goat serum for 1 h. Primary antibodies were applied and incubated overnight at 4°C. Following this, the cells were treated with FITC‐ or Cy3‐conjugated secondary antibodies (1:200) at 37°C for 60 min, and the slides were mounted using an agent containing 4′,6‐diamidino‐2‐phenylindole (Beyotime) for 5 min. F‐actin staining was conducted with phalloidin (Solarbio, Beijing, China) following the manufacturer's protocol. Fluorescent images were acquired using a fluorescence microscope (Olympus, Tokyo, Japan).

### 4.9 Western Blot Analysis

Total protein from NP cells was extracted using RIPA lysis buffer (Solarbio) supplemented with 1% phenylmethanesulfonyl fluoride and 1% phosphatase inhibitor (Solarbio). Protein concentrations were determined using the BCA Protein Assay Kit (Beyotime). Proteins were separated by SDS‐PAGE, transferred to polyvinylidene fluoride membranes, and blocked with blocking buffer (Beyotime). Membranes were incubated with primary antibodies overnight at 4°C, followed by incubation with HRP‐conjugated secondary antibodies at room temperature for 2 h. After washing with TBST, protein signals were detected using enhanced chemiluminescence (Bio‐Rad Lab., Hercules, CA, USA) and analyzed with ImageJ software (http://rsb.info.nih.gov/ij/) as previously described (Yan et al. 2024). The antibodies used in this study are listed in Table S2.

### 4.10 RNA Extraction and qRT‐PCR Array

Total RNA was isolated from NP cells using TRIzol reagent (TaKaRa, Shiga, Japan) according to the manufacturer's protocol. Reverse transcription was performed using the PrimeScript RT Reagent Kit (TaKaRa), and gene expression was assessed using TB Green PCR Master Mix (TaKaRa). The expression of genes involved in the MAPK signaling pathway was analyzed using a rat MAPK signaling pathway PCR array (Wcgene Biotech, Shanghai, China) following the manufacturer's instructions. Differential gene expression was quantified using the $2^{-\Delta \Delta C_{t}}$ method, with fold changes > 2 or < −2 considered biologically significant. The results were visualized using the Xiantao platform (https://www.xiantaozi.com).

### 4.11 SA‐β‐Gal Staining

The SA‐β‐gal activity was assessed using an SA–β‐gal staining kit (Beyotime) following the manufacturer's guidelines. Brightfield images were acquired using an Olympus MA BX53 microscope, and quantification of the stained cells was performed using ImageJ software (National Institutes of Health, Bethesda, MD, USA).

### 4.12 Lentivirus Transfection

Lentiviruses expressing short hairpin RNA (shRNA) targeting BRD4 (sh‐BRD4), MAP2K7 (sh‐MAP2K7), and PGF (sh‐PGF), along with scrambled control shRNA (sh‐NC) and lentiviruses overexpressing BRD4 (LV‐BRD4), MAP2K7 (LV‐MAP2K7), PGF (LV‐PGF), and a control lentivirus (LV‐NC) were purchased from GeneChem (Shanghai, China). NP cells were prepared at a density of 1–3 × 10^4^ cells/mL. The virus was introduced at a multiplicity of infection of 50, with the virus volume calculated as: (multiplicity of infection × number of cells)/viral titer. HiTransG P transfection enhancer was added, and the mixture was incubated for 8–12 h. After incubation, the medium was replaced with complete culture medium, and regular medium changes were made every 2–3 days. Cells were passaged at 80%–90% confluency for subsequent experiments.

### 4.13 Animal Experiments and Histological Evaluation

A puncture‐induced IDD model was established using healthy SD rats. The animals were randomly divided into four groups: Sham, IDD, IDD + sh‐NC, and IDD + sh‐BRD4. Anesthesia was induced using isoflurane and pentobarbital (40 mg/kg, intraperitoneally). A sterile incision was made to expose the Co7/8 intervertebral disk, followed by NP puncture using a 21‐G syringe needle. The needle was rotated 360° and left in place for 1 min. Lentiviral injections were performed with a microsyringe, and the operator was blinded to the treatment groups.

Eight weeks post‐surgery, rats were euthanized via pentobarbital overdose, and spine specimens were collected. The spines were fixed in 4% paraformaldehyde for 1 week, decalcified in ethylenediaminetetraacetic acid solution for 4 weeks, and embedded in paraffin. Midsagittal sections (5 μm) were prepared for histological staining, including H&E, Safranin O/Fast Green, Alcian blue, and Masson's trichrome staining (G1340; Solarbio). Staining procedures followed the manufacturer's instructions, and the stained sections were visualized and captured using a microscope (Olympus).

### 4.14 X‐Ray and MRI Assay

Rats were selected at random for radiography and MRI prior to euthanasia. Lateral radiographs were obtained using the DRX‐Ascend system (Siemens, Munich, Germany) with the following settings: current, 571 mA; voltage, 89.8 kV. The relative heights of the IVDs were measured as previously described. Prone‐position MRI was performed using a 3.0 T system (Philips Medical Systems, Best, The Netherlands) to obtain T2‐weighted images (repetition time, 1999.9 ms; echo time, 93.4 ms; slice thickness, 1.0 mm). The degree of IDD was assessed using the Pfirrmann classification, NP area, and gray value in a double‐blinded manner.

### 4.15 Immunohistochemistry

Paraffin‐embedded NP tissues were sectioned at 5 μm. Sections were deparaffinized, rehydrated, and subjected to antigen retrieval. Endogenous peroxidase activity was blocked using 3% hydrogen peroxide for 30 min. The sections were incubated overnight at 4°C with the following primary antibodies: BRD4 (1:200), P16 (1:100), P21 (1:100), IL‐6 (1:150), IL‐8 (1:150), MMP3 (1:100), MMP13 (1:100), collagen II (1:100), and aggrecan (1:100). Antibodies were detected using an immunohistochemistry kit (Maixin Biotech Co., Fuzhou, China) and the DAB Peroxidase Substrate kit (Maixin Biotech Co.) according to the manufacturer's protocols.

### 4.16 Statistical Analysis

All experiments were performed at least six times. Data are expressed as mean ± standard deviation (SD). For comparisons between two groups, a two‐tailed Student's t‐test was used. Multiple group comparisons were analyzed using one‐way analysis of variance. Correlation analysis was conducted using Spearman's correlation analysis. All statistical analyses were performed using GraphPad Prism 9.3.0 software (GraphPad Software, San Diego, CA, USA). Statistical significance was defined as follows: *p < 0.05; **p < 0.01; ***p < 0.001; ns, not significant.

## Author Contributions

G.Z., X.K., and L.L. designed experiments; G.Z., L.L., Z.Y, and Z.C. performed experiments; G.Z., X.H., and X.K. contributed to the writing – original draft preparation; G.Z., Y.W., and X.K. contributed to the writing – review and editing; G.Z. contributed to the visualization. All authors have read and approved the article.

## Conflicts of Interest

The authors declare no conflicts of interest.

## Supporting information


Figure S1.

Figure S2.

Appendix S1.

## Data Availability

All the data supporting the results of the present study are provided to the corresponding authors upon request.
