# Supplementary figures and images for: BRD4/MAP2K7/PGF Signaling Axis Promotes Senescence and Extracellular Matrix Metabolism of Nucleus Pulposus Cells in Intervertebral Disk Degeneration
Source: Aging Cell. 2025 Mar 25;24(6):e70034. doi: 10.1111/acel.70034 (PMC12151915; doi:10.1111/acel.70034)

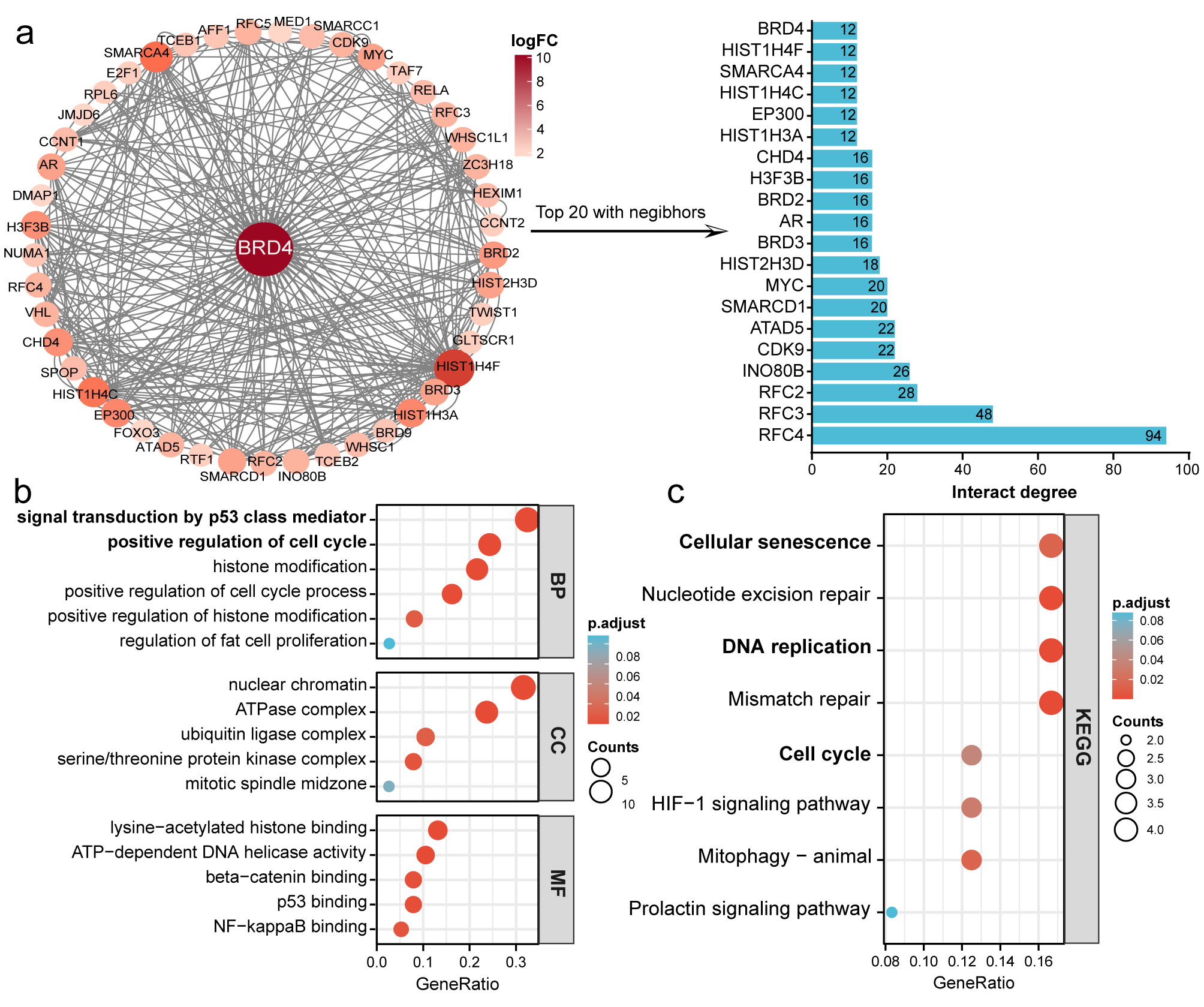

Supplement: Supplementary file 1 — Figure S1. [file ACEL-24-e70034-s001.tif]

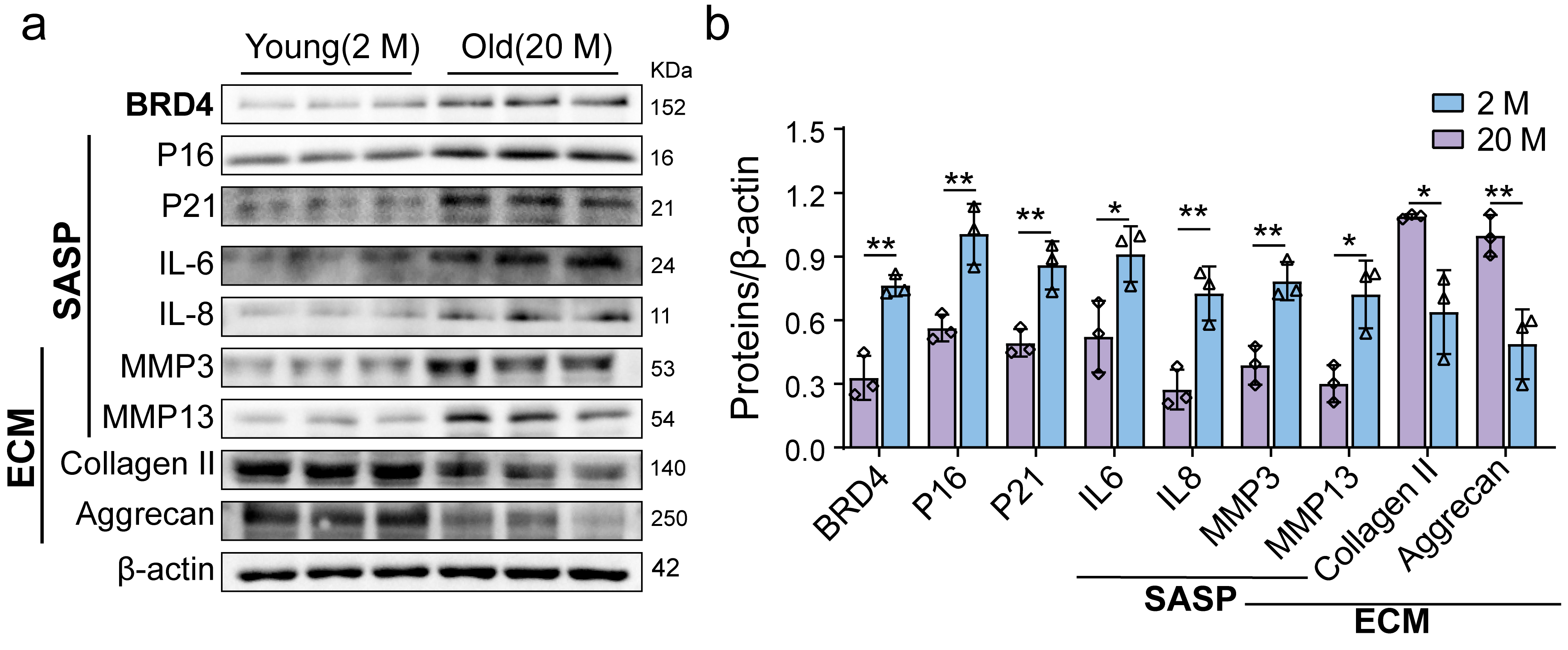

Supplement: Supplementary file 2 — Figure S2. [file ACEL-24-e70034-s002.tif]
