# Supplementary material for: BRD4/MAP2K7/PGF Signaling Axis Promotes Senescence and Extracellular Matrix Metabolism of Nucleus Pulposus Cells in Intervertebral Disk Degeneration
Source: Aging Cell. 2025 Mar 25;24(6):e70034. doi: 10.1111/acel.70034 (PMC12151915; doi:10.1111/acel.70034)
Supplement: Supplementary file 3 — Appendix S1. [file ACEL-24-e70034-s003.docx]

Supplementary Materials for

BRD4/MAP2K7/PGF signaling axis promotes the senescence and extracellular matrix metabolism of nucleus pulposus cells in intervertebral disc degeneration

Guangzhi Zhang^1,2,3,4,a^，Lei Li^1,2,3,4,a^，Zhili Yang^1,2,3,4,a^，Zhengyu Cao^1,2,3,4,a^，Xuchang Hu^1,2,3,4^，Yonggang Wang^1,2,3,4^，Xuewen Kang^1,2,3,4^✉

Correspondence to: ery_kangxw@lzu.edu.cn

**This PDF file includes:**

Figures. S1 to S2

Tables S1 to S3

Supplementary Fig. 1


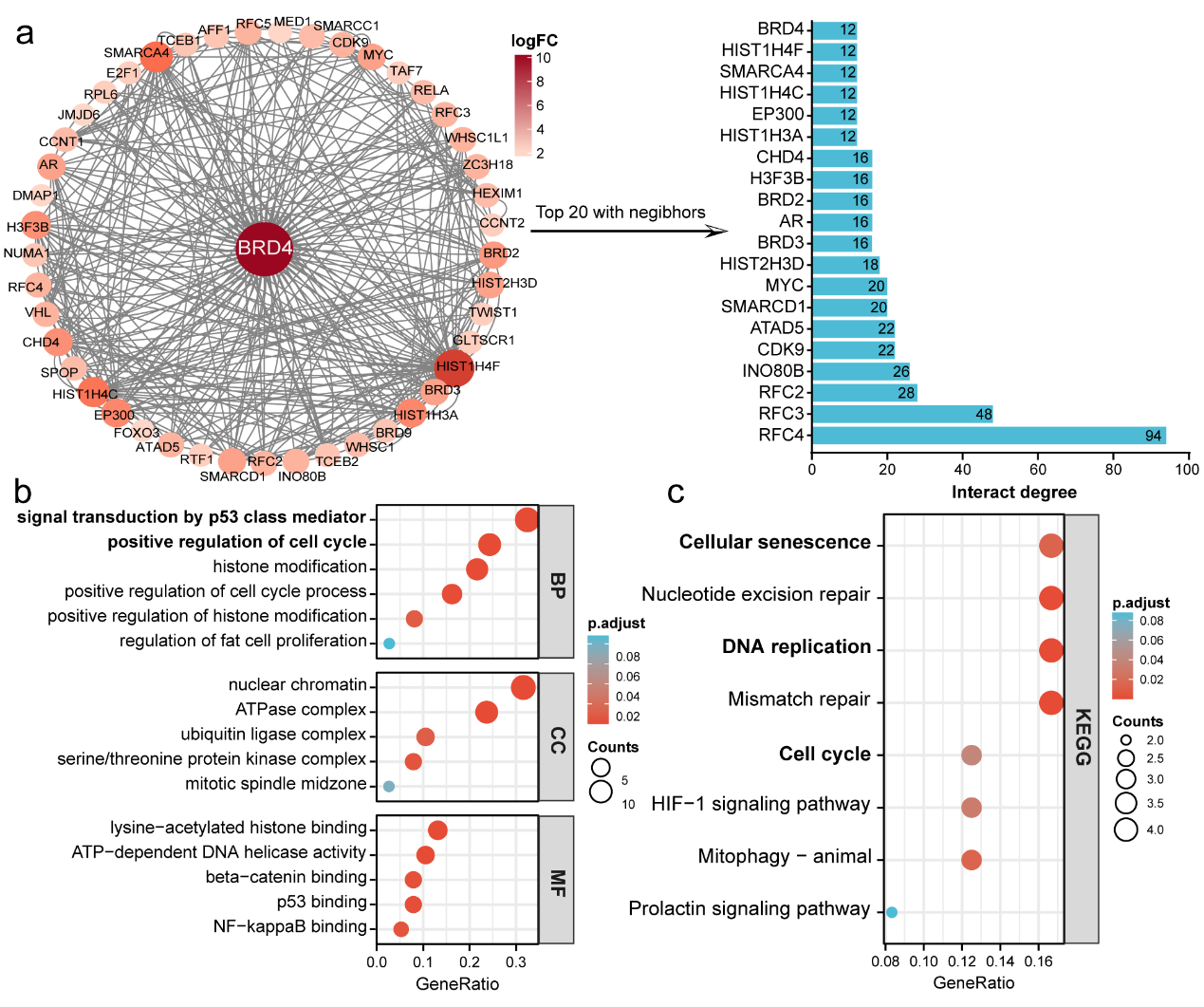


Supplementary Fig. 1 Construction of the BRD4 co-expression gene network and Gene Ontology (GO) and Kyoto Encyclopedia of Genes and Genomes (KEGG) enrichment analysis. (a) BRD4 co-expression gene network and the top 20 ranked genes. Using the STRING database with species set to 'Homo sapiens' and a confidence threshold >0.4, we obtained the BRD4 co-expression gene network data, which was then imported into Cytoscape for network visualization. The top 20 ranked genes include RFC4, RFC3, RFC2, INO80B, CDK9, ATAD5, SMARCD1, MYC, HIST2H3D, BRD3, AR, BRD2, H3F3B, CHD4, HIST1H3A, EP300, HIST1H4C, SMARCA4, HIST1H4F, and BRD4. (b) GO enrichment analysis of BRD4 co-expressed genes, including biological process (BP), cellular component (CC), and molecular function (MF). Colors closer to red indicate smaller P-values. (c) KEGG enrichment analysis of BRD4 co-expressed genes. Colors closer to red indicate smaller *P*-values.

Supplementary Fig. 2


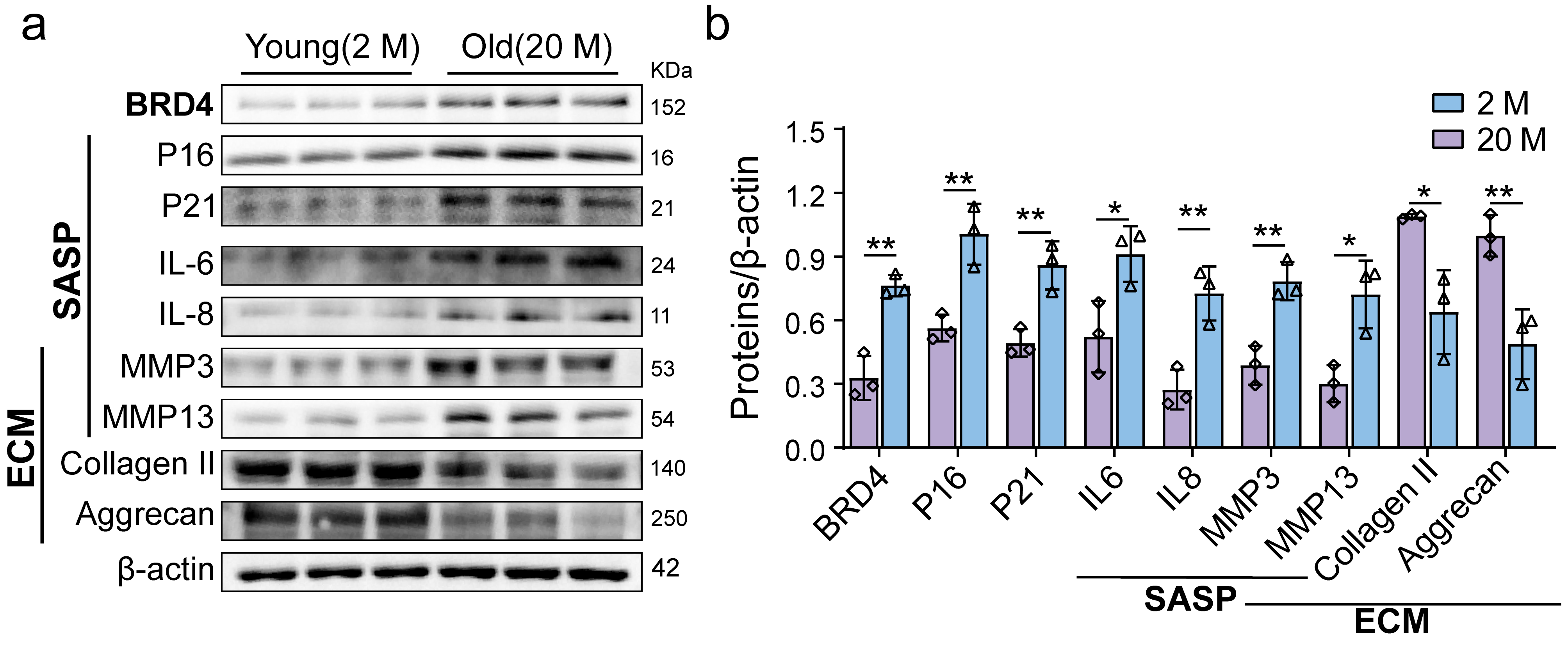


**Supplementary Fig. 2 BRD4 regulated cellular senescence and ECM metabolism in NP tissues of age-related IDD models in SD rats.** (a, b) Western blot bands and statistical analysis histograms of BRD4 expression, age-related proteins (P16 and P21), SASP markers (IL-6, IL-8, MMP3, and MMP13), and ECM metabolism indicators (MMP3, MMP13, Collagen II, and Aggrecan) in NP tissue of different age groups (2-month-old (2 M) and 20-month-old (20 M)) rats. **P*<0.05, ***P*<0.01.

**Supplementary Table 1. BRD4 co-expression gene network and GO and KEGG enrichment analysis.**

| Ontology | ID | Description | GeneRatio | BgRatio | pvalue | p.adjust | qvalue |
| --- | --- | --- | --- | --- | --- | --- | --- |
| BP | GO:0072331 | signal transduction by p53 class mediator | 12/37 | 267/18670 | 7.71e-14 | 1.10e-10 | 7.20e-11 |
| BP | GO:0045787 | positive regulation of cell cycle | 9/37 | 389/18670 | 5.01e-08 | 1.09e-05 | 7.12e-06 |
| BP | GO:0016570 | histone modification | 8/37 | 454/18670 | 2.39e-06 | 1.02e-04 | 6.67e-05 |
| BP | GO:0090068 | positive regulation of cell cycle process | 6/37 | 298/18670 | 2.41e-05 | 5.37e-04 | 3.52e-04 |
| BP | GO:0031058 | positive regulation of histone modification | 3/37 | 90/18670 | 7.48e-04 | 0.009 | 0.006 |
| BP | GO:0070344 | regulation of fat cell proliferation | 1/37 | 10/18670 | 0.020 | 0.088 | 0.058 |
| CC | GO:1904949 | ATPase complex | 9/38 | 101/19717 | 2.43e-13 | 2.55e-11 | 1.54e-11 |
| CC | GO:0000790 | nuclear chromatin | 12/38 | 377/19717 | 3.47e-12 | 1.82e-10 | 1.10e-10 |
| CC | GO:1902554 | serine/threonine protein kinase complex | 3/38 | 88/19717 | 6.47e-04 | 0.005 | 0.003 |
| CC | GO:0000151 | ubiquitin ligase complex | 4/38 | 282/19717 | 0.002 | 0.011 | 0.007 |
| CC | GO:1990023 | mitotic spindle midzone | 1/38 | 13/19717 | 0.025 | 0.077 | 0.046 |
| MF | GO:0070577 | lysine-acetylated histone binding | 5/38 | 19/17697 | 3.95e-10 | 2.55e-08 | 1.29e-08 |
| MF | GO:0004003 | ATP-dependent DNA helicase activity | 4/38 | 21/17697 | 1.05e-07 | 1.70e-06 | 8.59e-07 |
| MF | GO:0002039 | p53 binding | 3/38 | 66/17697 | 3.81e-04 | 0.001 | 7.53e-04 |
| MF | GO:0008013 | beta-catenin binding | 3/38 | 82/17697 | 7.20e-04 | 0.003 | 0.001 |
| MF | GO:0051059 | NF-kappaB binding | 2/38 | 29/17697 | 0.002 | 0.005 | 0.003 |
| KEGG | hsa03430 | Mismatch repair | 4/24 | 23/8076 | 5.12e-07 | 5.73e-05 | 4.25e-05 |
| KEGG | hsa03030 | DNA replication | 4/24 | 36/8076 | 3.32e-06 | 1.86e-04 | 1.38e-04 |
| KEGG | hsa03420 | Nucleotide excision repair | 4/24 | 47/8076 | 9.83e-06 | 3.67e-04 | 2.72e-04 |
| KEGG | hsa04137 | Mitophagy - animal | 3/24 | 68/8076 | 0.001 | 0.015 | 0.011 |
| KEGG | hsa04218 | Cellular senescence | 4/24 | 156/8076 | 0.001 | 0.015 | 0.011 |
| KEGG | hsa04066 | HIF-1 signaling pathway | 3/24 | 109/8076 | 0.004 | 0.032 | 0.023 |
| KEGG | hsa04110 | Cell cycle | 3/24 | 124/8076 | 0.006 | 0.040 | 0.029 |
| KEGG | hsa04917 | Prolactin signaling pathway | 2/24 | 70/8076 | 0.018 | 0.088 | 0.065 |

Supplementary Table 2. The list of primary antibodies.

| **Name** | **Company** |
| --- | --- |
| Anti-β-actin | Zhongshan Golden Bridge Biotechnology |
| Anti-BRD4 | Abcam |
| Anti-P16 | Abclonal Technology |
| Anti-P21 | Affinity Biosciences |
| Anti-IL-6 | ABclonal |
| Anti-IL-8 | Abmart |
| Anti-MMP3 | Affinity Biosciences |
| Anti-MMP13 | Affinity Biosciences |
| Anti-Aggreecan | Affinity Biosciences |
| Anti-Collagen II | Affinity Biosciences |
| Anti-SOX9 | Affinity Biosciences |
| Anti-MAP2K7 | Affinity Biosciences |
| Anti-PGF | Affinity Biosciences |

Supplementary Table 3. Clinical data of 24 patients with NP tissue samples.

| Specimen Number | Age (years) | Sex | Clinical diagnosis | Surgical site | Pfirrmann grades |
| --- | --- | --- | --- | --- | --- |
| 1 | 22 | female | lumbar disc herniation | L4/5 | II |
| 2 | 25 | male | lumbar disc herniation | L4/5 | II |
| 3 | 28 | male | lumbar disc herniation | L4/5 | II |
| 4 | 35 | male | lumbar disc herniation | L4/5 | II |
| 5 | 40 | male | lumbar disc herniation | L4/5 | II |
| 6 | 25 | female | lumbar disc herniation | L4/5 | II |
| 7 | 23 | male | lumbar disc herniation | L4/5 | II |
| 8 | 29 | male | lumbar disc herniation | L4/5 | II |
| 9 | 25 | female | lumbar disc herniation | L5/S1 | II |
| 10 | 48 | female | lumbar disc herniation | L4/5 | II |
| 11 | 37 | female | lumbar disc herniation | L4/5 | II |
| 12 | 46 | male | lumbar disc herniation | L4/5 | II |
| 13 | 55 | female | lumbar disc herniation | L4/5 | V |
| 14 | 48 | female | lumbar disc herniation | L5/S1 | V |
| 15 | 52 | male | lumbar disc herniation | L4/5 | V |
| 16 | 65 | female | lumbar disc herniation | L4/5 | V |
| 17 | 63 | female | lumbar disc herniation | L4/5 | V |
| 18 | 62 | male | lumbar disc herniation | L4/5 | V |
| 19 | 47 | male | lumbar disc herniation | L4/5 | V |
| 20 | 57 | male | lumbar disc herniation | L4/5 | V |
| 21 | 50 | male | lumbar disc herniation | L4/5 | V |
| 22 | 65 | male | lumbar disc herniation | L4/5 | V |
| 23 | 69 | male | lumbar disc herniation | L4/5 | V |
| 24 | 61 | male | lumbar disc herniation | L4/5 | V |
